# Species Interactions Determine the Importance of Response Diversity for Community Stability to Pulse Disturbances

**DOI:** 10.1111/ele.70299

**Published:** 2025-12-31

**Authors:** Charlotte Kunze, Owen L. Petchey, Shyamolina Ghosh, Helmut Hillebrand

**Affiliations:** ^1^ Institute for Chemistry and Biology of the Marine Environment (ICBM), School of Mathematics and Science Carl von Ossietzky Universität Oldenburg Oldenburg Germany; ^2^ Institute of Biodiversity Friedrich Schiller University Jena Jena Germany; ^3^ German Centre for Integrative Biodiversity Research (iDiv) Halle‐Jena‐Leipzig Leipzig Germany; ^4^ Department of Evolutionary Biology and Environmental Studies University of Zurich Zürich Switzerland; ^5^ Theoretical Sciences Visiting Program Okinawa Institute of Science and Technology Graduate University Onna Japan; ^6^ ECORISK Research Training Group Osnabrück University Osnabrück Germany; ^7^ Agricultural and Ecological Research Unit, Biological Science Division Indian Statistical Institute Kolkata West Bengal India; ^8^ Helmholtz‐Institute for Functional Marine Biodiversity at the University of Oldenburg (HIFMB) Oldenburg Germany; ^9^ Alfred‐Wegener‐Institute, Helmholtz Centre for Polar and Marine Research Bremerhaven Germany

## Abstract

Communities can buffer environmental change through diverse responses of their species, often leading to greater stability than expected from individual species. Metrics such as response dissimilarity (variation in magnitude) and divergence (variation in direction) capture this response diversity in fluctuating environments. We test whether response diversity also stabilises community properties under pulse disturbance. Combining model simulations of multi‐species communities with empirical data from a meta‐analysis, we find that community stability was consistently determined by the species mean response, regardless of interaction strength. Contrastingly, response dissimilarity and divergence were only related to stability in the absence of interspecific interactions. While response diversity increases stability under fluctuating conditions, pulse disturbances cause negative responses in most species and stability is highest when species uniformly exhibit strong resistance or fast recovery. These results highlight that the role of response diversity in promoting community stability depends on disturbance regimes and is shaped by species interactions.

## Graphical abstract

Caculations of response diversity can be based on species fundamental responses in isolation or their realised responses in the community using metrics of response dissimilarity and response divergence. Combining model simulations and a meta‐analysis, we show here that community stability in the context of pulse disturbance is primarily determined by the mean species response based on both realised and fundamental species responses, with response diversity contributing in the absence of interspecific interactions. In contrast to fluctuating environments, stability under pulse disturbances depends on species with strong resistance or rapid recovery, highlighting that both disturbance regime and species interactions shape stability outcomes.

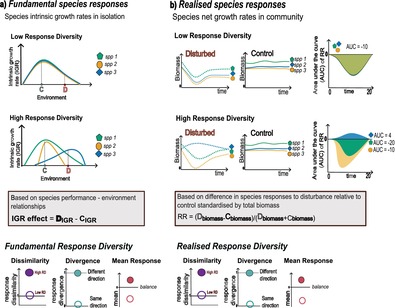

## 1 Introduction

As Earth's ecosystems face unprecedented and often human‐induced changes, predicting how ecological communities will respond to changing environmental conditions has become a major scientific challenge. Ecological stability captures the ability of an ecosystem to withstand and recover from disturbances (Pimm 1984) and is a multidimensional concept. It encompasses a variety of different metrics (Grimm and Wissel 1997; Ives and Carpenter 2007; Pimm 1984) that have been sorted (Donohue et al. 2013, 2016), decomposed (Hillebrand et al. 2018) and recently reassembled into an integrative metric of Overall Ecological Vulnerability (OEV) (Urrutia‐Cordero et al. 2021). Stability can be measured at the level of individual species and of emergent properties of the whole community, such as total biomass.

Community properties frequently exhibit greater stability than the properties of their component species (Tilman 1996, 1999). Hereby, diverse communities fluctuate less than species‐poor ones (‘insurance hypothesis’, Yachi and Loreau 1999) because they have a greater chance of harbouring species that are resistant to disturbances or because higher richness allows higher asynchrony of species abundances (Hautier et al. 2014; Loreau and de Mazancourt 2013). Hence, the buffering of community fluctuations can be based on mechanistic replacements (compensatory dynamics) but also increases the chance for different species responses by chance (‘portfolio effect’, Doak et al. 1998).

Elmqvist et al. (2003) described this asynchrony and difference in species responses to disturbances as response diversity. Unlike functional diversity, which focuses on the variation in traits that constrain species' roles in an ecosystem, response diversity specifically captures the variation in species' responses to change. Suding et al. (2008) introduced the terms ‘effect traits’ and ‘response traits’. Effect traits, such as maximum resource uptake rates, affect the rate of biomass production and thus influence ecosystem processes. They are associated with functional diversity. Response traits, such as the maximum temperature tolerance, constrain the response to temperature change. They inform about how species respond to environmental changes and are thus linked to response diversity. Under changing environmental conditions, the stability of functional properties can rely on both functional redundancy (multiple occurrence of the same effect trait) and response diversity, that is, multiple response traits of functionally similar species (Lawton and Brown 1994; Suding et al. 2008; Yachi and Loreau 1999). High response diversity enables compensatory dynamics, where declines in one species' biomass are offset by other species' increases because of their differing environmental responses (Gonzalez and Loreau 2009; Micheli et al. 1999; Mori et al. 2013).

Empirical evidence on how response diversity affects stability is scarce as the advancement of the theoretical concept has outpaced the empirical test of it (Ross and Sasaki 2023). Indeed, the majority of response diversity studies have been conceptual rather than analytical. Most empirical studies have measured response diversity as a response variable for stability of ecosystems but not as a predictor of stability (Ross et al. 2023). Of the studies empirically linking response diversity to stability of aggregate community properties (biomass or abundance), those conducted in fluctuating environments have often highlighted a positive effect of response diversity on temporal stability of biomass at the community level when response diversity was assessed directly from species response traits (Hordley et al. 2021; Leary and Petchey 2009; Ross et al. 2023). Other studies lacked this relationship between response diversity and temporal stability (Ross and Sasaki 2023), such as a study on pollination assessing response diversity as the difference in species' abundance or pollination service to land cover (Cariveau et al. 2013), reflecting the importance of environmental context.

Studies exploring the consequences of pulse disturbances find contrasting relationships between response diversity and resistance as well as resilience (recovery rate after disturbance, Glossary in Appendix S1, Table S1) (Baskett et al. 2014; Bhaskar et al. 2017). For example, Bhaskar et al. (2017) found that secondary forest recovery rates after a hurricane were slower with increasing response diversity, measured as variation in slopes of species‐specific response along a precipitation gradient. However, response diversity was positively related to resistance in this study, emphasising the often‐observed trade‐off between resistance and resilience, that is, recovery rate (Donohue et al. 2013; Hillebrand et al. 2018; Hillebrand and Kunze 2020). Despite these advances, the role of response diversity for ecological stability under pulse disturbances lacks both theoretical and empirical testing.

There is also the question of what information to use for measuring response diversity. One option is to derive response diversity from fundamental responses of species in isolation (Brennan and Collins 2015; Leary and Petchey 2009; Ross et al. 2023). Alternatively, it could be derived from realised responses in the presence of other species, which include interaction‐mediated effects of environmental change (Baskett et al. 2014; Bhaskar et al. 2017; Cariveau et al. 2013). Fundamental species responses are often the basis for modelling approaches, but their empirical application is constrained by the need to isolate and study each species individually—an approach that is often impractical and/or complex. Moreover, the applicability of fundamental species responses may be low where species interactions occur. In contrast, realised species responses may offer a more precise depiction of real‐world conditions (Gonzalez and Loreau 2009; Lajaaiti et al. 2024). Nevertheless, realised responses may depend strongly on the community context. More specifically, species' realised responses are shaped by competition, facilitation and other types of interspecific interaction as interaction strength increases, leading to different realised growth rates than expected from fundamental species responses to the environment. For instance, a species that experiences a weak negative effect of a pulse disturbance can nevertheless increase in abundance if its competitors experience strong negative effects of the disturbance, that is, there is competitive release. Likewise, strongly impacted species may recover faster when competitors are simultaneously suppressed. In summary, competitive interactions can change the impacts of disturbance across species by amplifying or dampening the effect of a pulse disturbance (Lajaaiti et al. 2024), making realised responses most suitable in the community context in which they are measured.

Theoretical (Ives et al. 1999; Ives and Cardinale 2004) and empirical studies (Ruiz‐Moreno et al. 2024) collectively suggest that response diversity plays a more important role in driving community dynamics than interspecific interactions. By contrast, evidence from a meta‐analysis indicates that altered species interactions may have a greater impact on population distribution and abundance than the direct effects of climate change (Ockendon et al. 2014). This leaves a knowledge gap about the influence of species interactions on the role of response diversity for community stability and about the influence of using fundamental or realised species responses.

Here, we ask (i) whether response diversity is related to community stability under pulse disturbances, (ii) whether the relationship between response diversity and stability differs when response diversity is derived from species' fundamental responses versus realised responses and (iii) whether this relationship depends on the strength of interspecific competition. We use two complementary approaches: mathematical simulations of competitive Lotka‐Volterra dynamics and empirical results obtained from a meta‐analysis of pulse‐disturbance experiments to test three hypotheses.

*Increasing response diversity increases the stability of functional properties of the community after pulse disturbance. Empirical data only allow deriving response diversity from realised responses*—*that is, in the presence of other species and under an unknown average interaction strength*—*whereas simulations provide insight into response diversity based on both fundamental and realised species responses under varying competition strengths*.

*The effect of response diversity differs between fundamental and realised responses. The effect of response diversity is higher when based on realised responses, as fundamental responses capture species' intrinsic sensitivities to environmental change in isolation, whereas realised responses integrate interspecific interactions effects*.

*The effect of response diversity on community stability depends on the strength of interspecific interactions. This is because, as interactions intensify, species' realised responses become increasingly shaped by interactions with other species, which can either buffer or amplify individual species' responses to disturbance*.

## 2 Methods

### 2.1 Simulated Communities

We simulated multi‐species communities using a discrete‐time version of the classical Lotka–Volterra model (De Mazancourt et al. 2013) with temperature‐dependent intrinsic growth rate and carrying capacity We created communities with 10 species differing in their optimum temperature for birth ($b_{\mathrm{opt},i}$), drawn from a uniform distribution with defined mean and range (Appendix S1 contains the model description). Intraspecific interactions were fixed ($\alpha_{\mathrm{ii}}=1$) whereas interspecific competition coefficients ($\alpha_{\mathrm{ij}}$) were drawn from a left‐skewed normal distribution with varying standard deviation.

#### 2.1.1 Pulse Disturbance

Each community experienced two independent simulations comprising a control run and a pulse disturbance. Simulations ran for 750‐time steps, including a 500‐step equilibration period at control temperature (22°C). In the pulse treatment, temperature decreased from 22°C to 15°C at time step 500, continued at 15°C for 50 time steps before returning to 22°C. Control simulations remained constant at 22°C. Only data from timepoint ≥ 500 contribute towards the analysis.

#### 2.1.2 Response Diversity and Interaction Strength

Response diversity was manipulated by systematically varying the mean and range of species temperature optima in a community, that is, $b_{\mathrm{opt},\text{mean}}$ and $b_{\mathrm{opt},\text{range}}$ (Appendix S1 Table S2). For example, communities with high values of $b_{\mathrm{opt},\text{mean}}$ contained species with high temperature optimum and were therefore mostly negatively affected by a pulse disturbance to lower temperature. Communities with high values of $b_{\mathrm{opt},\text{range}}$ contained species with very different temperature optima, so that some might respond positively and some negatively. Effects of competition strength were examined by systematically varying the average interaction strength of communities (by varying the value of ${\alpha_{\mathrm{ij}}}_{\mathrm{sd}}$). Each combination of ${\alpha_{\mathrm{ij}}}_{\mathrm{sd}},$ $b_{\mathrm{opt},\text{mean}}$ and $b_{\mathrm{opt},\text{range}}$ was replicated five times, giving a total of 14,175 independent communities.

### 2.2 Meta Analysis of Empirical Communities

We extracted species‐specific information from a published meta‐analysis on the effect of pulse disturbances on community stability (Hillebrand and Kunze 2020). Additionally, we updated the meta‐analysis based on a search at the Web of Science (www.webofknowledge.com/WOS, assessed July 16th, 2025) using the same search terms as for the original analysis ‘(experiment* or manipulat* or mesocosm* or microcosm*) AND recover* AND (disturb* or perturb* or pulse) AND (communit* or composit* or diversit* or assembl*)’. From the 110 publications of the original analysis and the 467 results of the new search, we retrieved those experiments that fulfilled the following selection criteria:

1. The study comprised of a disturbed treatment and an undisturbed control.
2. The disturbance was a pulse treatment inflicted upon a community
3. The study contained data on genus or species level.
4. At least 3 time points were sampled in the experiments

These criteria led to a database comprising 134 experiments from 44 publications (Appendix S2). For each time point we obtained means for the available univariate response variable (abundance/biomass) for control and treatment. Most studies reported abundance and only ~1/3 reported biomass values. Due to the large variation in the groups of organisms and the duration of the experiments, we converted the duration of the experiments to a scale of 0–1, with 1 being the last sampling date (Hillebrand and Kunze 2020).

### 2.3 Community (In‐)Stability to Disturbances

To assess community stability, we used the Overall Ecological Vulnerability (OEV), a metric of instability (Urrutia‐Cordero et al. 2021). The OEV is calculated as the area under the curve (AUC) of community biomass relative to an undisturbed control. In this framework, the AUC is calculated based on the total response of the disturbed community (Treat.Tot) normalised to the community response in control conditions (Con.Tot), as the response ratio of this difference (tot.RR).

$$\mathrm{tot}.\mathrm{RR}=\frac{\left(\text{Treat}.\mathrm{Tot}-\mathrm{Con}.\mathrm{Tot}\right)}{\left(\text{Treat}.\mathrm{Tot}+\mathrm{Con}.\mathrm{Tot}\right)}$$

$$\mathrm{OEV}=\mathrm{AUC}\left(\mathrm{tot}.\mathrm{RR}\right)$$

The OEV encompasses several dimensions of post‐disturbance stability and directly correlates with the overall effect size of the disturbance: A larger OEV indicates greater variability, weaker resistance to disturbance, slower recovery (resilience) and/or incomplete recovery (for overviews of these stability dimensions see Donohue et al. 2013; Hillebrand et al. 2018; Urrutia‐Cordero et al. 2021). In contrast to the original OEV, we consider both negative and positive deviations, allowing us to explore the relationship between community stability and realised species responses, which can differ by showing negative or positive deviations from control.

### 2.4 Species Responses to Pulse Disturbances

To assess the effect of the disturbance on each species, we standardised species‐specific responses to the disturbance with their response in control conditions. We distinguish between species *fundamental responses* and species *realised responses* to pulse disturbances. Species *fundamental responses* reflect their fundamental niche‐based responses to disturbance obtained from their performance along an environmental gradient in isolation. Specifically, species *fundamental responses* are determined as the change in intrinsic growth rate (IGR) between undisturbed (control) conditions and disturbed conditions, that is, the IGR effect. The intrinsic growth rate represents the intrinsic rate of population increase, defined as the difference between the temperature‐dependent birth rate $\left(b_{0,i}\right)$ and death rate $\left(d_{0,i}\right)$.

$$\mathrm{IGR}\;\text{effect}={\mathrm{IGR}}_{\text{disturbed}}-{\mathrm{IGR}}_{\text{control}}$$

The IGR effect becomes negative when the intrinsic growth rate in the disturbed environment is smaller than in the control and positive when the intrinsic growth rate is larger in the disturbed environment than in the control. Fundamental responses are thus independent of species interaction strengths.

Species *realised responses* reflect the difference in the net responses of species to a disturbance within the community relative to an undisturbed control, incorporating both the sensitivity of individual species to disturbances and the outcomes of interactions. Specifically, species realised responses are assessed from species biomass/abundance in the disturbed community relative to their biomass/abundance in the undisturbed control community. We base our calculation on the OEV instability metric, which is also the basis for our calculation of community stability, and when transferred to population level responses, reflects species' absolute contribution to stability (Kunze et al. 2024). Specifically, realised responses are determined as the AUC of the biomass response ratio, that is, the difference in species‐specific biomass in treatment (Treat.spp) and in control (Con.spp) divided by the summed biomass of the same species in treatment and control for each single time point:

$$\mathrm{RR}=\frac{\left(\text{Treat}.\mathrm{spp}-\mathrm{Con}.\mathrm{spp}\right)}{\left(\text{Treat}.\mathrm{spp}+\mathrm{Con}.\mathrm{spp}\right)}$$

This scaling constrains realised responses between −1 and 1 at each time step. If RR equals zero, the species is unaffected by disturbance at this time point. Negative RR reflects biomass declines; positive RR reflects biomass increases. The AUC of this deviation (AUC.RR) directly correlates with the overall disturbance effect on species in the community, that is, their *realised responses*. Negative values indicate a disadvantageous effect of the disturbance on the species over time by decreasing biomass production, whereas positive values indicate a beneficial effect of the disturbance by facilitating biomass production compared with the control.

In summary, we distinguish here between *fundamental* and *realised* responses (Appendix S1, Box 1). Fundamental responses reflect an absolute difference in species intrinsic growth rates in a disturbed environment relative to an undisturbed control. Realised responses represent an integrated, normalised difference in standing biomass in a community context, capturing the cumulative deviation in species absolute biomass over time. We calculated realised responses based on species net growth, given that most experiments in our meta‐analysis did not include the information needed for other approaches. Instead of the dichotomy between disturbed and control, we would need an environmental gradient to obtain slopes of species' realised responses (e.g., Bhaskar et al. 2017). Alternatively, we would need complete mono‐culture information alongside the community experiment to calculate fundamental species responses and use these weighted by species proportion in the community (e.g., Polazzo et al. 2025).

BOX 1 **Response diversity in a pulse disturbance context.**

Response diversity, that is, the asynchrony and difference in species responses to disturbances (Elmqvist et al. 2003), can be measured in several ways. These include the two measures of response dissimilarity and response divergence (Ross et al. 2023), alongside the mean species response (Table 1). However, we are aware that the mean response is not always independent of response diversity in our simulations (Appendix S1, Figure S2).

**TABLE 1:** Overview and interpretation of response diversity metrics, that is, dissimilarity and divergence and the mean species response.

Metric	Defintion	Formula and description	Interpretation	Source
Response dissimilarity	The overall magnitude of response diversity among species' responses	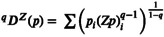 where q—sensitivity parameter that was set to zero (Ross et al. 2023), $p_{i}$– species responses, that is, fundamental responses (IGR_effect) and realised responses (AUC of effect size RR) Z—dissimilarity matrix based on pairwise Euclidean distances among species responses and ${\left(\mathrm{Zp}\right)}_{i}$‐ the ordinariness of the ith species within the community, that is, how similar each species' response is to others in the community where ${\left(\mathrm{Zp}\right)}_{i}=\sum_{j=1}^{s}Z_{\mathrm{ij}}p_{i}$ Pairwise Euclidean distances among species responses were used to construct the dissimilarity matrix Z, which was then incorporated into the Hill‐number framework to quantify response dissimilarity	1 < *y* < 2 *y* = 1, all species respond identically *y* = 2, species responses are as dissimilar as possible	Equation (1) in Leinster and Cobbold (2012), with modifications as described Ross et al. (2023)
Response divergence	The extent to which species declines are offset by gains	$\frac{\left(\max\left[x\right]-\min\left[x\right]\right)-\|\max\left[x\right]\left|-\right|\min\left[x\right]\|}{\left(\max\left[x\right]-\min\left[x\right]\right)}$ Where *x*—species responses, that is, fundamental responses (IGR_effect) and realised responses (AUC of the effect size RR) at a given environmental state relative to an undisturbed control Comparison of the range of species responses to the range of their absolute values quantifies directional variation across an environmental gradient. Instead of calculating the first derivatives of species performance‐environment relationships and assessing their interspecific variation (Ross et al. 2023), we evaluate the divergence between species' responses to disturbance relative to the control	0 < *y* < 1 *y* = 0 responses are identical, that is all response values are negative or all are positive *y* = 1 responses are dissimilar, and the most negative value is equal in magnitude to the most positive value	Ross et al. (2023)
Mean response	The mean response of species in a community to the disturbance	$\frac{\sum_{i=1}^{n}p_{i}}{n}$ $\text{where}\;p_{i}$– species responses, that is, fundamental responses (IGR_effect) and realised responses (AUC of the effect size RR)	y∈ℝ *y* < 0 species are negatively affected on average *y* > 0 species are positively affected on average	

**FIGURE 1:**
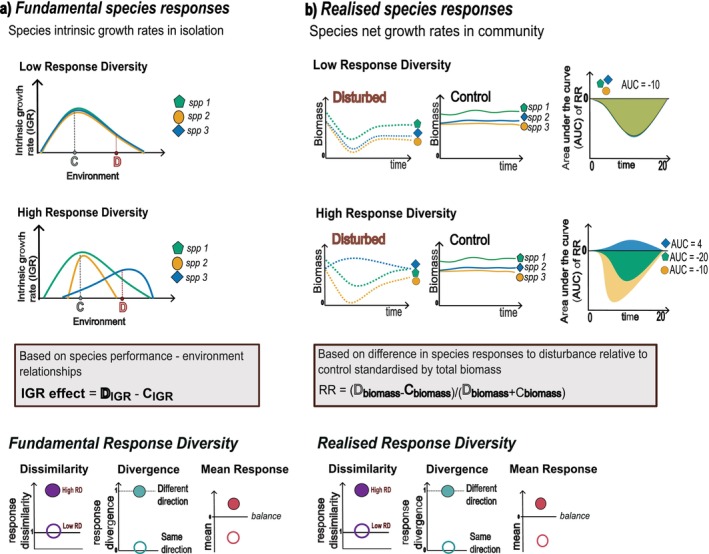
Exemplary calculation of response diversity metrics and the mean from fundamental species responses (a) and realised responses (b) for both low and high response diversity communities. Based on species fundamental reaction norms along an environmental gradient, species responses to a disturbance can be assessed as the effect on their intrinsic growth rate (IGR) (left). In comparison, species realised responses in the community can be assessed as the area under the curve (AUC) of the response ratio (RR), given by the difference in species responses to the disturbance (Dbiomass) relative to the control (Cbiomass). Response diversity can be assessed using the two consecutive measures: Response dissimilarity and divergence of fundamental species responses in isolation (fundamental response diversity) and their realised responses in the community (realised response diversity). Dissimilarity is low when species responses are identical, and high when species responses are dissimilar. Response divergence is low when all responses are either negative or positive, and high when the most negative response value is equal in magnitude to the most positive value. The mean of species fundamental and realised responses gives an idea on the average effect of the disturbance on species, respectively. Positive and negative deviations from zero in the mean response hence indicate a positive or negative trend.

Here, response diversity was assessed using two complementary components: the dissimilarity metric and the divergence metric. Both measures of response diversity were assessed from fundamental species responses (IGReffect), that is, fundamental response diversity, and from species realised responses (AUC.RR), that is, realised response diversity (Figure 1). We chose dissimilarity and divergence because they capture key complementary aspects of response diversity—overall variation in species' environmental responses and the extent to which those responses offset each other—making them ecologically relevant for assessing stability under environmental change. Although for pulse disturbances, dissimilarity and divergence are positively correlated (Appendix S1, Figure S3).

The dissimilarity‐based metric captures the overall magnitude of response diversity among species responses (Leinster and Cobbold 2012). Specifically, pairwise Euclidean distances among species responses were used to construct the dissimilarity matrix Z, which was then incorporated into the Hill‐number framework to quantify response dissimilarity (Table 1). Dissimilarity is low when species responses are identical, and high when species responses are dissimilar but independent of the direction. A dissimilarity of one indicates that all species respond identically.

The divergence metric aims to quantify the extent to which species declines are offset by gains (Yachi and Loreau 1999), and considers whether responses differ in direction. Instead of calculating the first derivatives of species performance‐environment relationships and assessing their interspecific variation (Ross et al. 2023), we evaluate the divergence between species' responses to disturbance relative to the control (Table 1). Response divergence is bound between zero and one, where zero indicates that all response values are either negative or positive, and one indicates that the most negative value is equal in magnitude to the most positive value.

As for response diversity, species mean response was calculated from *realised* and *fundamental* responses. A mean of zero results from *balance* in the negative and positive responses, that is, the sum of the positive values equals the sum of the negative values. Positive and negative deviations from zero hence indicate a positive or negative trend in the mean response.

### 2.5 Statistical Analysis

All calculations and simulations were performed using R version 4.4.0 (2024‐04‐24) (R Core Team 2024). The meta‐analysis was performed using the ‘metafor’ package (Viechtbauer 2010), visualisations and data transformations were performed using the packages cowplot (Wilke 2020), tidyverse (Wickham et al. 2019), and ggpubr (Kassambara 2020). Realised responses were estimated using the auc() function of the MESS package (Ekstrøm 2022).

For analysis of the simulation results, we removed very rare species from the analyses (fewer than 10 entries with abundances higher than 10^−3^) to prevent overestimating the impact of very rare species for the calculation of realised responses.

In contrast to the simulations, empirical data from the meta‐analysis allowed only calculating realised species responses, as studies did not contain monospecific information, and thus fundamental responses could not be obtained. Community stability was assessed from the summed biomass/abundance of all species within a community analogous to the model simulations. To determine the relationship between response diversity and mean species responses on community stability, we performed an unweighted mixed‐effects meta‐analysis with community instability as response variable, mean species responses, response dissimilarity and divergence as moderators, and experimentID as random effect.

## 3 Results

### 3.1 Simulations

#### 3.1.1 Species' Fundamental Responses

**FIGURE 2:**
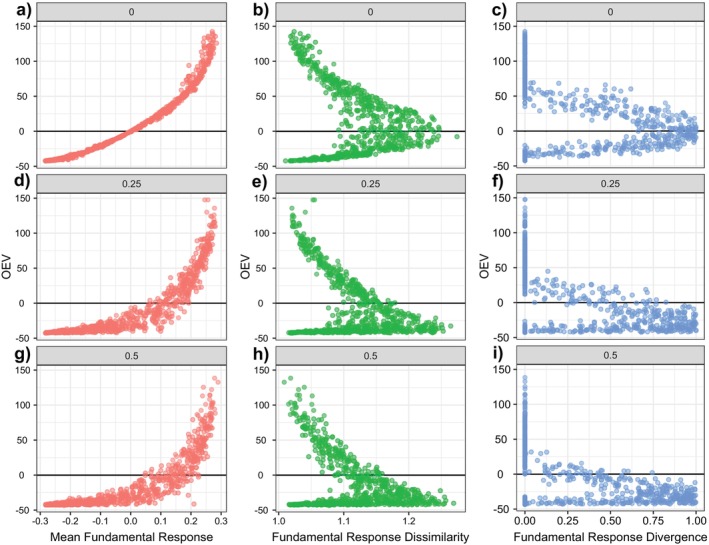
Community instability, calculated as the OEV, as a function of the mean fundamental response, fundamental response dissimilarity, and fundamental response divergence for communities without interactions (a–c), intermediate interaction strength (d–f) and high interaction strength (g–i). Community instability decreases for balanced mean fundamental responses (a) and increasing fundamental response diversity (b, c); however, this relationship weakened with increasing interaction strength (d–i). Each point represents one model community (subset of *n* = 2025 communities); average strength of species interactions (sd) increases from top to bottom row.

In the absence of interspecific interactions (Figure 2a–c), the simulations showed strong relationships between community instability (OEV) and the mean fundamental response as well as both metrics of fundamental response diversity. Instability was lowest when the mean response was zero, whereas positive or negative mean responses led to higher instability (Figure 2a) with the sign conserved, as treatments could deviate positively or negatively from control. Highest response diversity, measured as dissimilarity (Figure 2b) or divergence (Figure 2c), was associated to lowest instability (OEV≈0). Reductions in fundamental response diversity resulted in positive or negative OEV; the magnitude of instability (OEV) was correlated to the magnitude of response diversity (Figure 2b,c).

Adding interspecific competition of intermediate and high strength (Figure 2d–i) to the simulations resulted in similarly shaped relationships between OEV and mean response as well as response diversity but shifted towards more negative deviations in OEV. This shift towards more negative deviations in OEV with increasing response diversity is the result of interactions in our model simulations that are purely competitive and reduce the net growth rates of species. Specifically, increasing competition resulted in a 16% lower mean OEV. The mean response showed the same monotonic relationship as for fundamental responses with community instability when interaction strength increased (Figure 2a,d,g), but with increasing variability. Moreover, a more positive mean fundamental response was required for lowest instability (OEV≈0). Similarly, increasing interaction strength maintained the V‐shaped relationship between fundamental response dissimilarity and community OEV (Figure 2b,e,h) and between fundamental response divergence and community OEV, respectively (Figure 2c,f,i). However, highest response diversity was no longer associated with lowest instability, but negative OEV. Reductions in response diversity then led to even more negative OEV or less negative OEV turning positive. Lowest instability was consequently associated with intermediate response diversity.

#### 3.1.2 Species' Realised Responses

**FIGURE 3:**
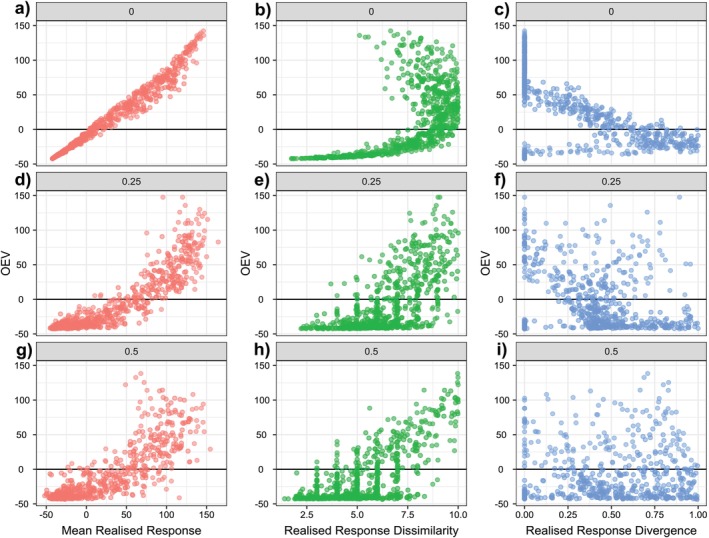
Community instability, measured as OEV, as a function of mean realised response, realised response dissimilarity and realised response divergence for no interactions (a–c), intermediate interaction strength (d–f), and high interaction strength (g–i). Community instability was low for balanced mean realised response (a) and increasing realised response diversity (b, c), but only without species interactions. Each point represents one model community (subset of *n* = 2025 communities); average strength of species interactions (sd) increases from top to bottom row.

In the absence of competition, the monotonic relationship between community instability and the mean realised response (Figure 3a) as well as the V‐shaped relationship to both metrics of realised response diversity (Figure 3b,c) mirrored the patterns observed for fundamental species responses (cf Figure 2a–c). However, the relationships were shifted such that lowest instability (OEV≈0) was observed at slightly positive mean realised responses (Figure 3a) and below the maximum response diversity (Figure 3b,c). This indicates that more positive responses were required to balance the negative disturbance effects.

Increasing competition strength led to greater heterogeneity in the relationship between response diversity measures and community instability (Figure 3d–i). The relationship between realised mean response and the OEV showed greater variation with increasing competition strength and a shift towards higher mean response for maximum stability (cf. Figure 3d,g with Figure 3a). While these results mirrored closely the analyses of the fundamental mean response (see above), the same comparison for response diversity metrics showed qualitative changes for metrics based on realised metrics. For realised response dissimilarity, the relationship turned monotonic with increasing competition strength (Figure 3b,e,h). Low response dissimilarity resulted in large negative OEV, increasing dissimilarity led to high variation in OEV, while high dissimilarity resulted in large positive OEV (Figure 3e,h). For response divergence, the V‐shaped relationship found in simulations without interactions (Figure 3c) gradually disappeared with increasing competition strength (Figure 3f,i).

### 3.2 Meta‐Analysis

**FIGURE 4:**
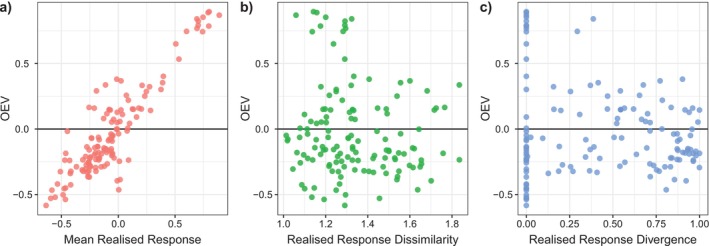
Community instability, measured as OEV, as a function of mean realised species responses (a), realised response dissimilarity (b) and realised response divergence (c). Community instability was low when realised responses were balanced (a), but was unrelated to realised response diversity metrics (b, c). Each point represents one community in the meta‐analysis (*n* = 134).

In the meta‐analysis, we only had access to realised responses and lacked information on the mean interspecific interaction strengths, as the original studies typically did not include monocultures or pairwise interaction data. Hence, we only assessed the relationship between realised response diversity and the mean response with community instability. Similar to the simulations, community instability was significantly related to the mean response, showing the same monotonic relationship than observed in the simulations (Figure 4a; Appendix S1 Table S3; Mixed‐effects model slope = 0.97 ± SE = 0.27, *p* < 0.01). Community instability (OEV) was not significantly related to measures of response diversity (Figure 4b,c; Appendix S1 Table S3; *p* > 0.05). Largest (positive or negative) OEV occurred at low response divergence, otherwise response diversity was not related to community OEV.

## 4 Discussion

Our results highlight the context‐dependent role of response diversity for community stability under pulse disturbances. This does not contradict its stabilising role under environmental fluctuations (Leary and Petchey 2009; Ross et al. 2023) but emphasises that these mechanisms do not translate easily to other types of environmental change. The stability of both simulated and empirical communities was consistently determined by mean response rather than response diversity, with the relationship between mean response and community stability shifted by competition strength in the simulations (rejecting H1). Community stability was related to the mean response regardless of whether it was derived from realised or fundamental responses (partly accepting H2). High response diversity resulted in high community stability in the absence of species interactions. However, the relationship between response diversity and community stability weakened with increasing interaction strength, with a stronger effect on realised responses than on fundamental responses (accepting H3). Combining a meta‐analysis and model simulations, our study provides mechanistic insights into the multifaceted role of response diversity for community stability in the context of pulse disturbances.

### 4.1 Response Diversity in a Pulse Disturbance Context

Our combined model simulations and meta‐analysis of experimentally disturbed communities revealed that, contrary to expectations, response diversity did not determine community stability under pulse disturbance (Figures 2 and 3). This contrasts with findings from fluctuating environments, where high response diversity typically enhances stability (Leary and Petchey 2009; Ross et al. 2023). Fluctuating environments are characterised by recurrent positive and negative deviations from a long‐term mean (Harris et al. 2018; Ives and Carpenter 2007). Here, high response diversity promotes stability as diverse species responses increase the likelihood of including species with environmental optima both above and below the mean, facilitating compensatory dynamics (Mori et al. 2013). As such, in fluctuating environments diverse species responses enable a community to buffer environmental changes and maintain functioning across varying conditions (Downing et al. 2008; Yachi and Loreau 1999).

Pulse disturbances, however, differ fundamentally in nature. Defined by their abrupt onset and short duration, they shift environmental conditions in a single direction (e.g., a temporary drop in temperature) before conditions recover (Donohue et al. 2016). Under such disturbances, species with optima above the mean increase response diversity, but are disproportionately negatively affected, ultimately reducing community stability. For example, a microcosm study on marine phytoplankton showed empirically that only species with high thermal optima contributed positively to community functioning during a heatwave (Bestion et al. 2020). In a terrestrial system, pollinator communities exposed to land‐use intensification exhibited thermal resilience largely because dominant fly species with cooler temperature preferences compensated for the decline of other taxa (Kühsel and Blüthgen 2015). Likewise, a long‐term study in kelp forests found that the stabilising role of biodiversity during marine heatwaves was contingent not on species richness per se, but on the functional identities of the constituent species (Liang et al. 2024). In a pulse disturbance context, stability thus relies less on diverse responses and more on the presence of a few species with high resistance or rapid recovery, allowing the community to maintain functionality if there is functional redundancy (Hillebrand et al. 2008; Hillebrand and Kunze 2020).

Community stability was consistently determined by the mean species response to pulse disturbance for both simulated and empirical communities. Stability required a balance in species responses—not just variation, but symmetry around zero, that is, equal variation above and below zero. This balance can emerge when all species show no response to the disturbance (low dissimilarity/divergence), all species respond differently (high dissimilarity/divergence) (Appendix S1, Figures S2, S3), or a mixture of these occurs, so long as responses are centred on zero. Similarly, one other study found that species coexistence did not depend on the variation in species responses but on the mean response across species, when analysing the size of the feasibility domain in response to pollution and warming for simulations and empirical macroinvertebrate communities (De Laender et al. 2023). Moreover, a recent study turned this ‘imbalance’ in species responses into a metric and showed that in fluctuating environments higher balance in species responses (lower imbalance) is related to greater temporal stability (Polazzo et al. 2025). Consequently, response diversity alone is not sufficient for stability; rather, the distribution and symmetry of species responses relative to zero play a crucial role.

The results of the meta‐analysis largely reflected the results of the simulations for realised responses, suggesting that the observed patterns are independent of the introduced disturbances but might occur because of the nature of the disturbance type. While our model simulations introduced a single pulse disturbance, comprising a drop in temperature, the meta‐analysis covers a wide range of disturbances such as pollution, species removal, drought, or flood and therefore represents an ideal test case.

### 4.2 The Influence of Interspecific Interaction Strength

Increasing competition strength weakened the relationship between both response diversity and the mean species response with community stability (Figures 3 and 4). In the absence of species interactions, increasing response diversity increases the likelihood of including species with optimal traits that allow species persistence for the given disturbance (Hillebrand et al. 2008; Yachi and Loreau 1999). Once interactions are introduced, species no longer respond solely based on their intrinsic traits, as interspecific interactions shape their realised responses (Lajaaiti et al. 2024). As a result, even species with optimal traits may experience reduced biomass or be outcompeted. Such competitive dynamics lead to a shift where stability was not related to highest response diversity because of lower asynchrony in species responses under pulse disturbance.

Indeed, increasing interaction strength led to more negative realised species responses, as species with higher T_opt_ and higher competitiveness showed greater biomass loss (Appendix S1, Figure S7). On the other hand, species with lower T_opt_ and higher competitiveness gained even more biomass because of their competitive advantage under pulse disturbance (temperature drop). These opposing patterns in species‐level responses suggest compensatory dynamics, where the competitive release allowed biomass losses in some species to be offset by biomass gains in others (Gonzalez and Loreau 2009; Micheli et al. 1999). Increasing interaction strength led to greater biomass losses of sensitive species and reductions in emergent community functions. Consequently, a higher mean species response, that is higher biomass production, is required to compensate for these losses and maintain community stability. In other words, compensatory dynamics do not result from high diversity in species responses alone but require the ability of some species to maintain or increase biomass production under increasing interspecific interactions (Ernest and Brown 2001).

Other dimensions of stability, that is resistance, resilience (rate of recovery), temporal stability and final recovery (Hillebrand et al. 2018), largely mirrored the patterns found for OEV (Appendix S1, Figures S4–S6). Resistance and recovery increased with mean species response in both simulations (with intermediate and strong competition) and empirical data, indicating that communities with positive mean performance suffered smaller biomass losses during the pulse. While an increase in (response) diversity increases the probability of including resistant species in the community, it also increases the chance for highly susceptible ones (Hillebrand et al. 2008). Moreover, the same increase in mean responses was associated with lower resilience, indicating that resistant communities tended to recover more slowly. Taken together, our results reflect the often observed trade‐off between resistance and resilience where a strong capacity to resist disturbance can constrain the potential for rapid recovery (Hillebrand and Kunze 2020).

In contrast to our meta‐analysis that showed no relationship between resistance and resilience with increasing response diversity, an empirical study on hurricane impacts reported a strong relationship between response diversity and resistance and resilience (Bhaskar et al. 2017). However, our simulations showed that interspecific competition can mask the effect of response diversity on dimensions of stability (e.g., resistance and resilience), leading to both positive and negative variations with increasing diversity (Appendix S1, Figures S5, S6). Indeed, another meta‐analysis found that altered species interactions have a greater impact in shaping population abundance than climate change effects (Ockendon et al. 2014). Other studies in fluctuating environments have shown that response diversity is more important than species interactions for community stability (Ives et al. 1999; Polazzo et al. 2025; Ruiz‐Moreno et al. 2024). In fact, the importance of response diversity for community stability may be determined by the intensity of environmental change (Mori et al. 2013). When environmental changes are extreme and particularly when coupled with species extinctions, response diversity may become more important for community stability than species interactions. Combined with our results, this highlights that the stabilising mechanisms of response diversity are context‐dependent and vary between different disturbance types.

### 4.3 Limitations and Recommendations for Future Studies

Here, we show the limitations of response diversity to explain community stability in response to pulse disturbances. In such pulse disturbance contexts, there is often a single favourable response that allows species persistence (Arnoldi et al. 2019; Hillebrand and Kunze 2020). By contrast, in fluctuating environments, maintaining stability over time requires high response diversity (Ross et al. 2023; Ross and Sasaki 2023). Given the increasing importance of extreme events with proceeding climate change (Bathiany et al. 2018; Harris et al. 2018), we see a pressing need to further explore the role of response diversity for disturbances besides fluctuating environments. Future studies should systematically compare different disturbance types (e.g., pulse, press and fluctuations) to better understand the generality of response diversity as a stability mechanism.

Given the apparent differences between realised and fundamental response diversity with increasing interaction strength, we show here the benefit of assessing response diversity and the mean response from both species' fundamental responses in isolation and their realised responses in community. The meta‐analysis was constrained to realised responses, as the original studies rarely reported monocultures or pairwise interaction data. This limits our ability to disentangle the impact of species sensitivities to disturbance on community stability from interspecific interactions that modulate species responses. In other words, for communities with unknown interactions, it is difficult to interpret patterns that emerge as communities change when exposed to disturbances. To better understand community stability to disturbance, a holistic approach including both species' realised responses and fundamental responses of species in isolation is advisable. This requires bottom‐up experiments that build up the level of complexity and allow explicit exploration of the role of interspecific interactions in shaping community stability. Specifically, it would be useful to include monoculture treatments and pairwise competition assays in experimental designs (e.g., Polazzo et al. 2025). Such designs would allow more direct tests of how response diversity obtained from fundamental and realised responses shape community stability under pulse disturbances.

Another important difference between model simulations and the meta‐analysis is the fact that we aimed for a wide range of response diversity in our model simulations by varying temperature optima distribution across species. By contrast, experiments underlying the meta‐analysis mostly used naturally co‐occurring species that might have a narrower diversity in species responses to disturbance. Experiments that systematically introduce varying levels of response diversity, while accounting for multiple stability metrics, will help to further elucidate the mechanisms underlying varying biodiversity–stability relationships (Pennekamp et al. 2018).

## 5 Conclusion

Here we show that the importance of response diversity for community stability is context dependent and differs depending on the prevailing strength of interspecific interactions. Although the mean species response, rather than existing metrics of response diversity, determined community stability in our study, this does not mean that a diverse set of species responses is not important for maintaining community stability under external forcing. Rather, it is not enough to have variation in responses, but there needs to be equal variation above and below zero for communities with no interactions. When interaction strengths are high, mean responses must also be higher to buffer the negative effects on community function in the face of pulse disturbance and thus provide the same benefit to stability. In a pulse disturbance context, there is often only one good response that allows species to persist and maintain functionality through disturbance (Arnoldi et al. 2019; Hillebrand and Kunze 2020). As increasing (response) diversity also increases the probability of containing resistant species (Hillebrand et al. 2008), it is essential to maintain as much biodiversity as possible to ensure ecosystem resilience to global change (Dee et al. 2017, 2019; Ives and Carpenter 2007).

## Author Contributions

C.K. and H.H. designed the study. C.K. collected the data and performed the meta‐analysis. S.G. wrote the model; C.K. and O.L.P. performed the model analyses. C.K. wrote the first draft of the manuscript. All authors contributed to manuscript drafts.

## Funding

This work was supported by Deutsche Forschungsgemeinschaft, HI848/29‐1, RTG 3004‐1; Ocean Floor Excellence Cluster, EXC2077; Schweizerischer Nationalfonds zur Förderung der Wissenschaftlichen Forschung, 10002183.

## Supporting information


**Appendix S1:** ele70299‐sup‐0001‐AppendixS1.docx. **Table S1**: Glossary of used terms.
**Figure S1:** Species abundances over time for one exemplary model run of simulated communities experiencing a pulse disturbance (dotted line) and control conditions (solid line). Different facets indicate the different species. The pulse disturbance consisted of a temperature decrease of 5°C at timepoint 500 respectively, whereafter the temperature returned to 20°C, the control stayed at 20°C constantly. For this model run, the mean temperature optimum of the community was at 19.5°C, competitive interactions were weak with alpha_ij_sd = 0.
**Table S1:** Overview of model parameters and variables.
**Figure S2:** Fundamental response diversity measures, that is fundamental response divergence (a) and fundamental response dissimilarity (b) as a function of the mean fundamental species response (a, b). Realised response dissimilarity (c) and realised response divergence (b) as a function of mean realised species response (c, d). Fundamental mean species responses were calculated as the mean intrinsic growth rate (IGR) effect of the disturbance. Different facets indicate average interaction strength in community, one point is one community (*n* = 2025).
**Figure S3:** Correlation between realised and fundamental response diversity measures, that is, response divergence and response dissimilarity, respectively. Realised response divergence and dissimilarity were weakly correlated (Spearman‐Rank correlation, *R* = 0.2, *p* < 0.01), while fundamental response divergence and dissimilarity were strongly correlated (Spearman‐Rank correlation, *R* = 0.88, *p* < 0.01). Different facets indicate average interaction strength in community, one point is one community (*n* = 2025).
**Figure S4:** Stability metrics of resistance (a), resilience (b), temporal stability (c) and recovery (d) as a function of realised response diversity measures and mean realised response in empirical communities from the meta‐analysis. Each dot represents one community (*n* = 134), different facets and colours indicate mean response (red), or realised response diversity metrics, that is, dissimilarity (green) and divergence (blue).
**Figure S5:** Stability metrics of resistance (a–c), resilience (d–f), temporal stability (g–i) and recovery (j–l) as a function of mean fundamental response and fundamental response diversity measures. Each dot represents one community (*n* = 121), different facets and colours indicate mean response (red), or fundamental response diversity metrics, that is, dissimilarity (green) and divergence (blue). Each point represents one model community (*n* = 1350), with intermediate (sd = 0.25) and strong competition (sd = 0.5).
**Figure S6:** Stability metrics of resistance (a–c), resilience (d–f), temporal stability (g–i) and recovery (j–l) as a function of mean realised response and realised response diversity measures. Each dot represents one community (*n* = 121), different facets and colours indicate mean response (red), or realised response diversity metrics, that is, dissimilarity (green) and divergence (blue). Each point represents one model community (*n* = 1350), with intermediate (sd = 0.25) and strong competition (sd = 0.5).
**Figure S7:** Realised species responses as a function of species competitiveness and their temperature optimum (in °C). For competitiveness, high values indicate high competitiveness on average. One point indicates one species (*n* = 2025). Different facets indicate strength of competition with higher values indicating stronger competition (alpha_ij_sd).
**Figure S8:** Fundamental species responses, calculated as the difference in intrinsic growth rate (IGR), as a function of species competitiveness and their temperature optimum (in °C). For competitiveness, high values indicate high competitiveness on average. One point indicates one species (*n* = 2025). Different facets indicate strength of competition with higher values indicating stronger competition (alpha_ij_sd).
**Table S3:** Results of the mixed effects meta‐analysis with community OEV, Resistance, Resilience, Temporal Stability (1/CV) and Recovery and absolute OEV as response variables respectively, and caseID random effect, mean species responses and response diversity metrics, dissimilarity and divergence, as moderators. Significant effects of moderators are indicated in bold (*n* = 134). Values have been rounded to the third decimal.

**Appendix S2:** ele70299‐sup‐0002‐AppendixS2.docx. **Figure S1:** PRISMA Flow for extending meta‐analysis. *Source:* Page MJ, et al. BMJ 2021; 372:n71. doi: 10.1136/bmj.n71. The original meta‐analysis was performed in 2018 based on a search at the Web of Science (www.webofknowledge.com/WOS, assessed April 3rd, 2018).

## Data Availability

Code for meta‐analysis and simulations is archived on Zenodo https://doi.org/10.5281/zenodo.17775521. Meta‐analysis data are published on Figshare: https://doi.org/10.6084/m9.figshare.28803887.
