# Supplementary material for: Species Interactions Determine the Importance of Response Diversity for Community Stability to Pulse Disturbances
Source: Ecol Lett. 2025 Dec 31;29(1):e70299. doi: 10.1111/ele.70299 (PMC12755191; doi:10.1111/ele.70299)
Supplement: Supplementary file 1 — Appendix S1: ele70299‐sup‐0001‐AppendixS1.docx. Table S1: Glossary of used terms. Figure S1: Species abundances over time for one exemplary model run of simulated communities experiencing a pulse disturbance (dotted line) and control conditions (solid line). Different facets indicate the different species. The pulse disturbance consisted of a temperature decrease of 5°C at timepoint 500 respectively, whereafter the temperature returned to 20°C, the control stayed at 20°C constantly. For this model run, the mean temperature optimum of the community was at 19.5°C, competitive interactions were weak with alpha_ij_sd = 0. Table S1: Overview of model parameters and variables. Figure S2: Fundamental response diversity measures, that is fundamental response divergence (a) and fundamental response dissimilarity (b) as a function of the mean fundamental species response (a, b). Realised response dissimilarity (c) and realised response divergence (b) as a function of mean realised species response (c, d). Fundamental mean species responses were calculated as the mean intrinsic growth rate (IGR) effect of the disturbance. Different facets indicate average interaction strength in community, one point is one community (n = 2025). Figure S3: Correlation between realised and fundamental response diversity measures, that is, response divergence and response dissimilarity, respectively. Realised response divergence and dissimilarity were weakly correlated (Spearman‐Rank correlation, R = 0.2, p < 0.01), while fundamental response divergence and dissimilarity were strongly correlated (Spearman‐Rank correlation, R = 0.88, p < 0.01). Different facets indicate average interaction strength in community, one point is one community (n = 2025). Figure S4: Stability metrics of resistance (a), resilience (b), temporal stability (c) and recovery (d) as a function of realised response diversity measures and mean realised response in empirical communities from the meta‐analysis. Each dot represen [file ELE-29-0-s002.docx]

**Appendix S1**

**Journal: Ecology Letters**

**Article Type: Letter**

**Title:** Species interactions determine the importance of response diversity for community stability to pulse disturbances

**Authors:** Charlotte Kunze, Owen L. Petchey, Shyamolina Ghosh, Helmut Hillebrand

**Table S1**: Glossary of used terms

| **Term** | **Definition** |
| --- | --- |
| AUC | Area under the curve |
| Disturbance | Changes to the biotic or abiotic environment that affect the structure and dynamics of ecosystems (White & Pickett, 1985). |
| Pulse disturbance | Discrete disturbance with a defined start and end. |
| Ecological Stability | Multidimensional concept capturing an ecosystem’s ability to absorb and recover from change, integrating metrics of resistance, resilience, recovery, and temporal variability (Donohue et al., 2013; Hillebrand et al., 2018; Kéfi et al., 2019; Urrutia‐Cordero et al., 2021). |
| Functional redundancy | Functional redundancy captures the idea that multiple species can perform overlapping ecological functions, so that if one species declines under disturbance, others can compensate and maintain community-level functioning (Lawton and Brown, 1994) |
| Fundamental species responses | Species responses to disturbance in isolation, measured here as the absolute difference in species intrinsic growth rates (IGR) in a disturbed environment relative to an undisturbed control (IGR effect). |
| Realised species responses | The net responses of species to a disturbance within the community relative to an undisturbed control. Realised species responses represent an integrated, normalized difference in standing biomass, capturing the cumulative deviation in absolute biomass over time. |
| Response diversity | Asynchrony and difference in species responses to disturbances (Elmqvist et al., 2003) |
| Resistance | Ability to withstand disturbance (Pimm, 1984). |
| Resilience | Ability to recover from disturbance; measured as the rate of recovery (Hillebrand et al., 2018; Pimm et al., 2019) |
| Recovery | The ability to return to undisturbed conditions following a disturbance (Hillebrand et al., 2018). |
| Temporal variability | Variability of functional properties over time, estimated as the coefficient of variation (CV). |
| OEV | Overall Ecological Vulnerability, an integrative metric of instability based on the area under the curve (Urrutia‐Cordero et al., 2021) |

**Model formulation**

We simulated multi-species community using a discrete-time version of the classical Lotka-Volterra model (De Mazancourt et al., 2013) with temperature-dependent intrinsic growth rate and carrying capacity. Population growth of species *i* is given as

$$r_{i}\left( t \right)=\ln N_{i}\left( t+1 \right)-\ln N_{i}\left( t \right)$$

$=r_{mi}[1-\frac{N_{i}\left( t \right)+\sum_{j \neq i} \alpha_{ij}N_{j}(t)}{K_{i}}]$ (Eq. 1)

where $N_{i}\left( t \right)$ corresponds to the biomass of species *i* at time *t.* The $r_{mi}$ is the temperature dependent intrinsic rate of natural increase, which is given by the difference between the birth rate $\left( b_{0,i} \right)$and death rate $(d_{0,i})$. $K_{i}$ is carrying capacity, and $\alpha_{ij}$, the interspecific competition coefficient describing the effect of species *i* on *j*. While *r* and *K* are temperature-dependent variables, $\alpha$ is independent of the environment.

The carrying capacity *K* of species *i* is linked to the intrinsic rate of natural increase $r_{mi}$ as

$K_{i}=\frac{r_{mi}}{\beta+\delta}$(Vasseur, 2020; Eq 14.6)

where $\beta$ and $\delta$ are the density dependent constants and $r_{mi}$ is the temperature dependent intrinsic rate of natural increase, given by the difference between the birth rate $\left( b_{0,i} \right)$and death rate $(d_{0,i})$.

Temperature dependence was incorporated into the birth and death rate of populations:

$$b_{0,i}\left( T \right)=abe^{\frac{{-\left( T-b_{opt,i} \right)}^{2}}{s_{i}}}$$

$d_{0,i}\left( T \right)=ade^{zT}$ (Vasseur, 2020; Eq 14.5)

where, $a$is an intercept, $b_{opt,i}$is the temperature at which intrinsic growth rate is highest, $s$ is the width of intrinsic growth rate-birth rate function, $z$ is the slope of death rate-temperature function and scales the effect of temperature on death rate (in °C) to mimic the Arrhenius relationship. Combining the birth rate temperature function and death rate temperature function gives the left-skewed shape common for temperature performance curves. Thus, a temperature disturbance directly alters population growth rates via its effect on birth and death rates.

Species' $b_{opt,i}$values were drawn from a uniform distribution with a specified mean and range (Table S2), generating communities with low, intermediate, or high thermal optima. Low mean values produced communities dominated by cold-adapted species; small ranges produced communities with similar temperature preferences (see Figure S1 for an exemplary model run).

Intraspecific interactions were fixed ($\alpha_{ij}$=1). Interspecific interactions ($\alpha_{ij})$were taken as absolute values of draws from a left-skewed normal distribution with mean zero and a specified standard deviation (Table S2). Small standard deviations generated weak competition; larger values produced strong competitive interactions.

**Fig. S1**: Species abundances over time for one exemplary model run of simulated communities experiencing a pulse disturbance (dotted line) and control conditions (solid line). Different facets indicate the different species. The pulse disturbance consisted of a temperature decrease of 5°C at timepoint 500 respectively, whereafter the temperature returned to 20 °C, the control stayed at 20 °C constantly. For this model run, the mean temperature optimum of the community was at 19.5 °C, competitive interactions were weak with alpha_ij_sd = 0.

**Table S1:** Overview of model parameters and variables.

| **Variable or parameter** | **Description** | **Value/ Formula** | **Reference** |
| --- | --- | --- | --- |
| **Species level variables and parameters** | | | |
| $\boldsymbol{r}_{\boldsymbol{i}}\left( \boldsymbol{t} \right)$ | Growth rate of species *i.* | $r_{i}\left( t \right)=\ln N_{i}\left( t+1 \right)-\ln N_{i}\left( t \right)$  $=r_{mi}[1-\frac{N_{i}\left( t \right)+\sum_{j \neq i} \alpha_{ij}N_{j}(t)}{K_{i}}]$ | (De Mazancourt et al., 2013) |
| $\boldsymbol{N}_{\boldsymbol{i}}\left( \boldsymbol{t} \right)$ | The biomass of species *i* at time *t.* |  |  |
| $\boldsymbol{r}_{\boldsymbol{m,i}}$ | Intrinsic rate of natural increase. defined by the birth and death rate | Birth rate minus death rate. |  |
| $\boldsymbol{b}_{\boldsymbol{0,i}}\left( \boldsymbol{T} \right)$ | Temperature-dependent birth rate with Gaussian function. | $b_{0,i}\left( T \right)=a_{b}e^{\frac{{-\left( T-b_{opt,i} \right)}^{2}}{s_{i}}}$ | (Vasseur, 2020; Eq 14.5) |
| $\boldsymbol{b}_{\boldsymbol{opt,i}}$ | Temperature optimum of birth rate of species *i* | Drawn from a uniform distribution with mean $\boldsymbol{b}_{\boldsymbol{opt,mean}}$and range $\boldsymbol{b}_{\boldsymbol{opt,range}}$ |  |
| $\boldsymbol{a}_{\boldsymbol{b,i}}$ | Birth rate at optimum temperature | 0.3; same for all species in all simulated communities. |  |
| $\boldsymbol{s}_{\boldsymbol{i}}$ | Width of temperature response curve of birth rate. | 10; same for all species in all simulated communities. |  |
| $\boldsymbol{d}_{\boldsymbol{0,i}}\left( \boldsymbol{T} \right)$ | Temperature-dependent death rate with Arrhenius function. | $d_{0,i}\left( T \right)=a_{d}e^{zT}$ | (Vasseur, 2020; Eq 14.5) |
| $\boldsymbol{a}_{\boldsymbol{d,i}}$ | Constant of temperature-death rate function | 0.1; same for all species in all simulated communities. | (Vasseur, 2020) |
| $\boldsymbol{z}$ | Slope of temperature-death rate function. | 0.05; same for all species in all simulated communities. |  |
| $\boldsymbol{K}_{\boldsymbol{i}}$ | Carrying capacity of species *i*. | $K_{i}=\frac{r_{mi}}{\beta+\delta}$ | (Vasseur, 2020; Eq 14.6) |
| $\boldsymbol{\beta, \delta}$ | Density dependent constants for species carrying capacity. | 0.001; same for all species in all simulated communities. |  |
| **Community level parameters** | | | |
| $\boldsymbol{\alpha}_{\boldsymbol{ij}}$ | The strength of interspecific interaction between species *i* and *j*. | Absolute value of draws from a normal distribution with mean 0 and standard deviation $\boldsymbol{\alpha}_{\boldsymbol{ij}}\boldsymbol{\_sd}$ |  |
| $\boldsymbol{\alpha}_{\boldsymbol{ij}}\boldsymbol{\_sd}$ | Strength of interspecific competition in a community. | There were 21 levels of interaction strength, ranging from 0-0.5 in steps of 0.025. Low values create a community with weak competitive interactions, high values create a community with strong competitive interactions. |  |
| $\boldsymbol{b}_{\boldsymbol{opt,mean}}$ | Mean interspecific value of optimum temperature.  ***Controls response diversity of a community.*** | There were 15 values of $\boldsymbol{b}_{\boldsymbol{opt,mean}}$ ranging from 15 to 22 in 0.5°C steps. Low values make a community of species with low temperature optima. |  |
| $\boldsymbol{b}_{\boldsymbol{opt,range}}$ | Range of interspecific optimum temperatures.  ***Controls response diversity of a community.*** | There were 9 values of $\boldsymbol{b}_{\boldsymbol{opt,range}}$ ranging from 3 to 7 in 0.5°C steps. Low values make a community of species with similar temperature optima. |  |
| ***S*** | Species richness | 10; same for all communities |  |
| ***T*** | Temperature | Control = 22°C Pulse = 15°C |  |


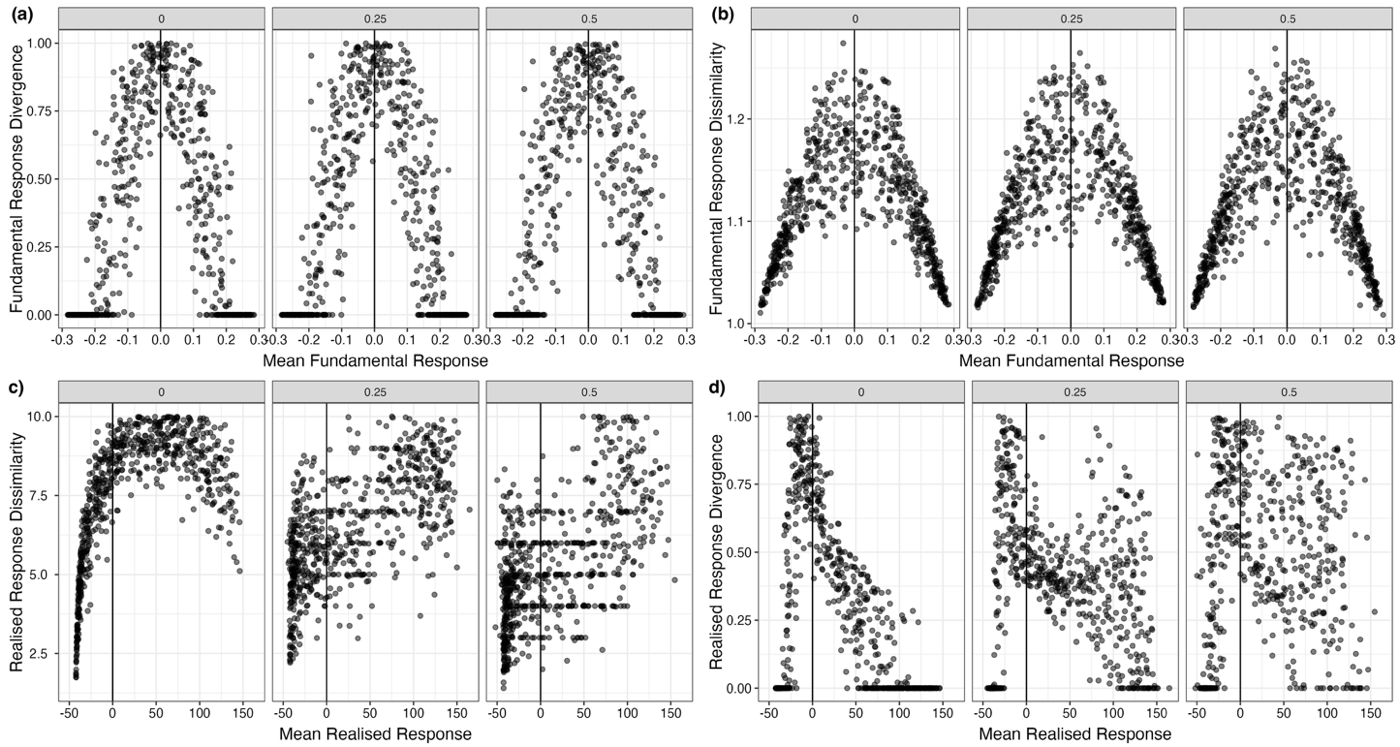


**Fig. S2:** Fundamental response diversity measures, that is fundamental response divergence (a) and fundamental response dissimilarity (b) as a function of the mean fundamental species response (a, b). Realised response dissimilarity (c) and realised response divergence (b) as a function of mean realised species response (c,d). Fundamental mean species responses were calculated as the mean intrinsic growth rate (IGR) effect of the disturbance. Different facets indicate average interaction strength in community, one point is one community (n = 2025).

*
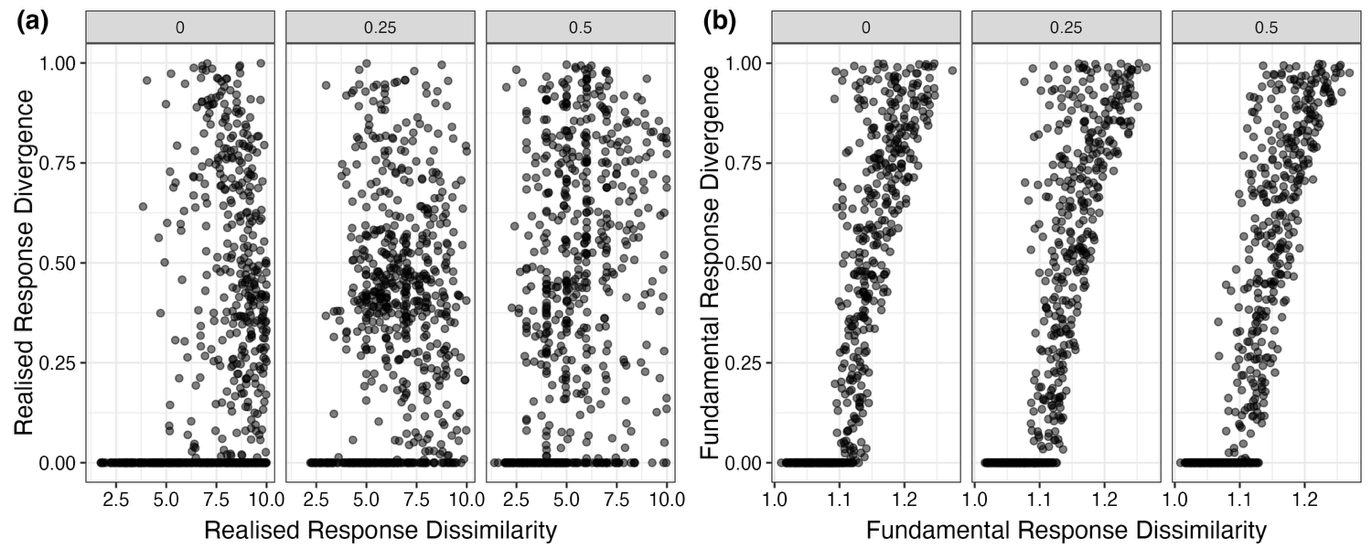
*

**Fig. S3:** Correlation between realised and fundamental response diversity measures, i.e. response divergence and response dissimilarity, respectively. Realised response divergence and dissimilarity were weakly correlated (Spearman-Rank correlation, R = 0.2, p<0.01), while fundamental response divergence and dissimilarity were strongly correlated (Spearman-Rank correlation, R =0.88, p-value <0.01). Different facets indicate average interaction strength in community, one point is one community (n = 2025).


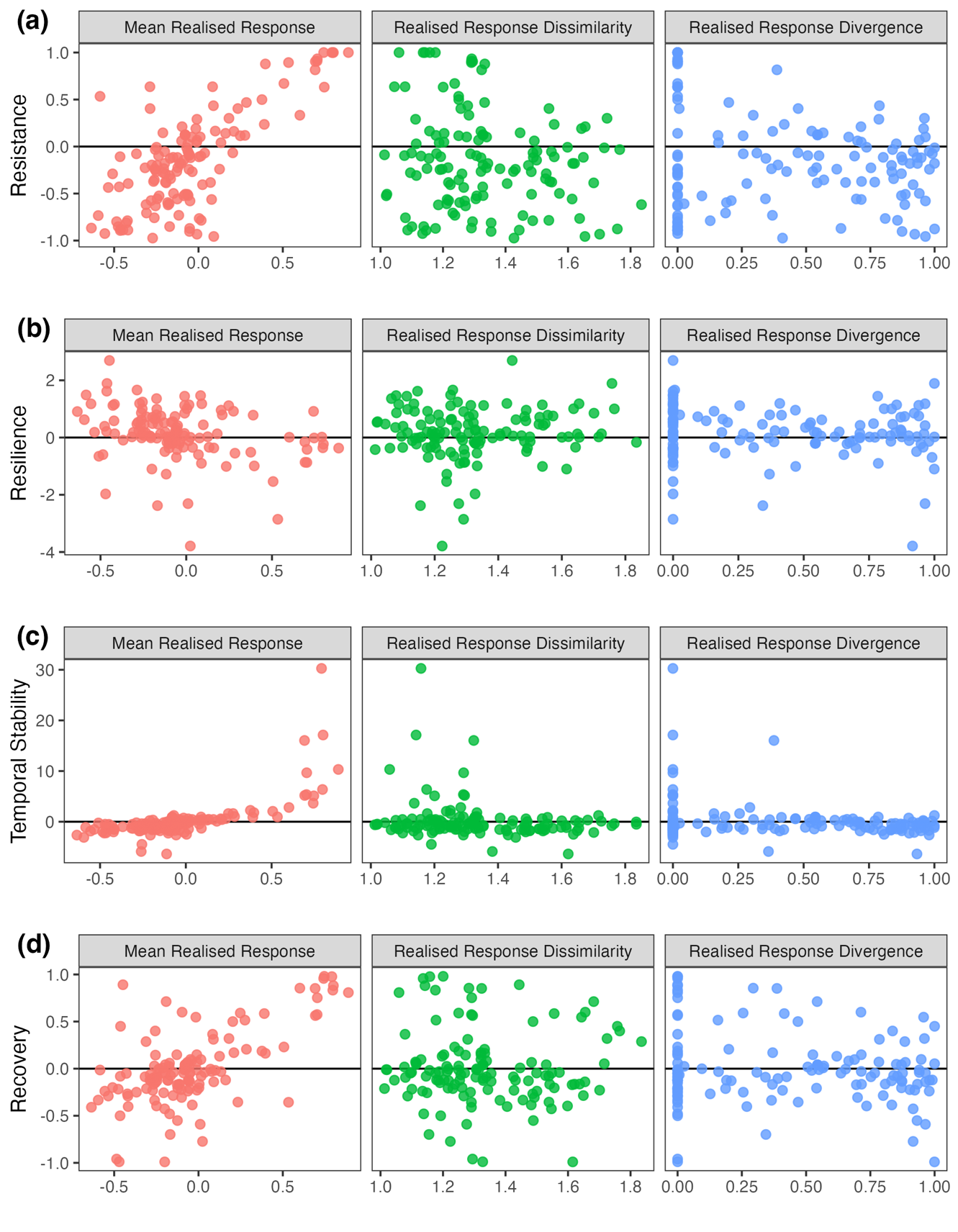


**Fig. S4:** Stability metrics of resistance (a), resilience (b), temporal stability (c), and recovery (d) as a function of realised response diversity measures and mean realised response in empirical communities from the meta-analysis. Each dot represents one community (n=134), different facets and colors indicate mean response (red), or realised response diversity metrics, i.e. dissimilarity (green) and divergence (blue).

*
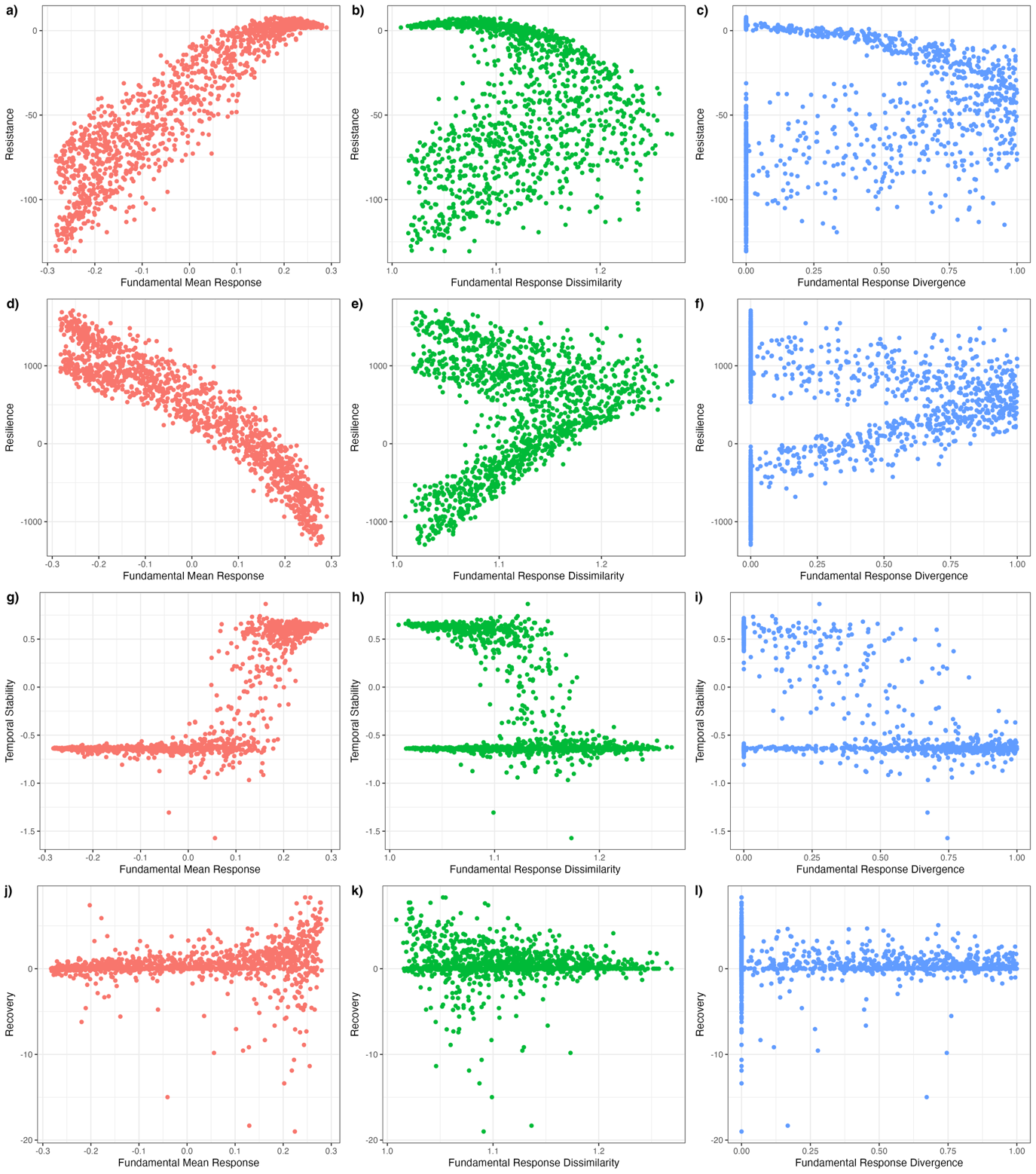
*

**Fig. S5:** Stability metrics of resistance (a-c),resilience (d-f), temporal stability (g-i), and recovery (j-l) as a function of mean fundamental response and fundamental response diversity measures. Each dot represents one community (n=121), different facets and colors indicate mean response (red), or fundamental response diversity metrics, i.e. dissimilarity (green) and divergence (blue). Each point represents one model community (n= 1350), with intermediate (sd = 0.25) and strong competition (sd =0.5). .


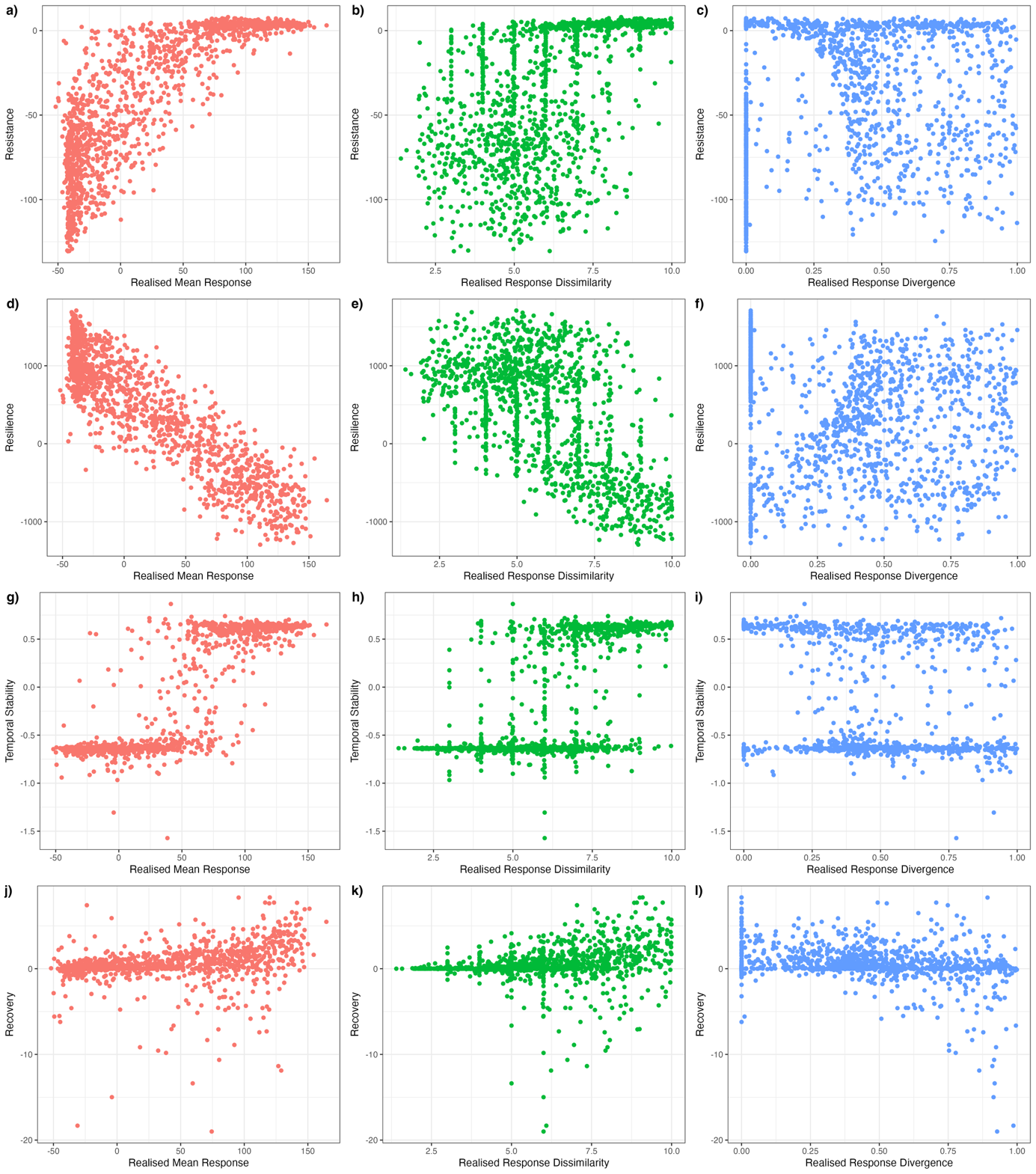


**Fig. S6:** Stability metrics of resistance (a-c),resilience (d-f), temporal stability (g-i), and recovery (j-l) as a function of mean realised response and realised response diversity measures. Each dot represents one community (n=121), different facets and colors indicate mean response (red), or realised response diversity metrics, i.e. dissimilarity (green) and divergence (blue). Each point represents one model community (n= 1350), with intermediate (sd = 0.25) and strong competition (sd =0.5). .


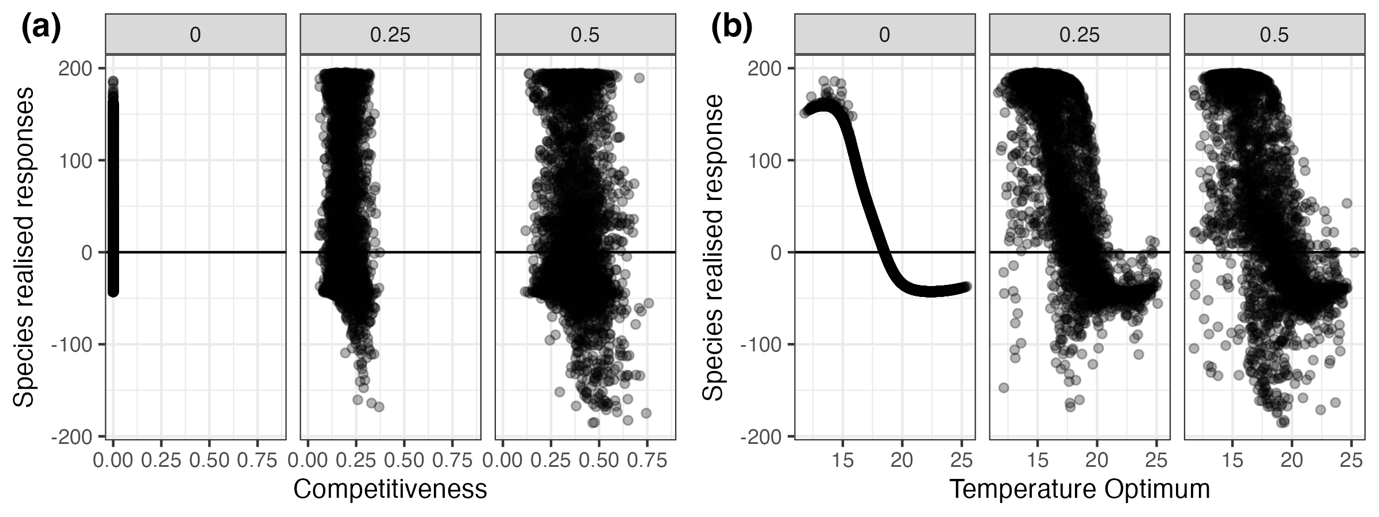


**Fig. S7:** Realised species responses as a function of species competitiveness and their temperature optimum (in °C). For competitiveness, high values indicate high competitiveness on average. One point indicates one species (n = 2025). Different facets indicate strength of competition with higher values indicating stronger competition (alpha_ij_sd).

*
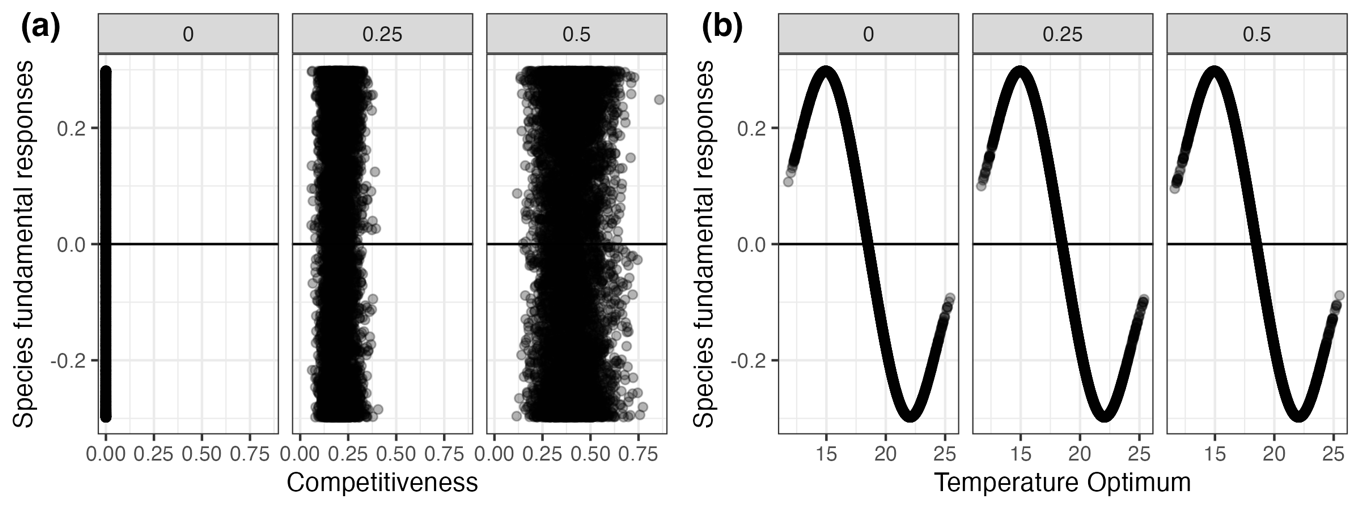
*

**Fig. S8:** Fundamental species responses, calculated as the difference in intrinsic growth rate (IGR), as a function of species competitiveness and their temperature optimum (in °C). For competitiveness, high values indicate high competitiveness on average. One point indicates one species (n = 2025). Different facets indicate strength of competition with higher values indicating stronger competition (alpha_ij_sd).

**Table S3**: Results of the mixed effects meta-analysis with community OEV, Resistance, Resilience, Temporal Stability (1/CV), and Recovery and absolute OEV as response variables respectively, and caseID random effect, mean species responses and response diversity metrics, dissimilarity and divergence, as moderators. Significant effects of moderators are indicated in bold (n = 134). Values have been rounded to the third decimal.

| **Moderat-or** | **Instability (OEV)** | | **Resistance** | | **Resilience** | | **Temporal Stability** | | **Recovery** | |
| --- | --- | --- | --- | --- | --- | --- | --- | --- | --- | --- |
|  | **esti-mate** | **p- value** | **Esti-mate** | **p- value** | **Esti-mate** | **p- value** | **Esti-mate** | **p- value** | **Esti-mate** | **p- value** |
| intercept | -0.1225 | 0.860 | -0.070 | 0.925 | -0.683 | 0.353 | 0.664 | 0.732 | -4.30 | 0.558 |
| Mean response | **0.967** | **<0.01** | **1.164** | **<0.01** | **-0.828** | **0.002** | **8.421** | **<0.01** | **0.783** | **<0.01** |
| Dissimi-larity | 0.153 | 0.789 | 0.044 | 0.942 | 0.745 | 0.218 | 0.636 | 0.689 | 0.437 | 0.469 |
| Diver-gence | -0.116 | 0.689 | -0.159 | 0.594 | -0.464 | 0.120 | **-2.101** | **<0.01** | -0.243 | 0.415 |

**Appendix Box 1: Comparing realised and fundamental species responses**

For calculation of species responses to pulse disturbances, we distinguish between fundamental responses and realised responses. Fundamental responses were derived from changes in intrinsic growth rate measured from species in isolation under disturbed versus control conditions:

IGReffect = IGR_disturbed_ – IGR_control_

Realised responses were calculated by integrating the difference in absolute species biomass between disturbed and control conditions, standardised by their summed biomass:

$Realised response =\int_{t=0}^{n} {RR}_{t} dt,$ where $RR= \frac{N_{t, Disturbed}-N_{t, Control}}{(N_{t, Disturbed}+N_{t, Control})}$

Standardisation by the sum ${(N}_{t, Disturbed}+N_{t, Control})$ controls for differences in absolute species biomass, such that species with very different total biomasses may exhibit comparable realised responses if their deviations from control remain small (AUC values; Box Fig.S1). This scaling constrains realised responses between –1 and 1 at each time step, producing a characteristic S-shaped relationship between realised and fundamental responses (Box1 Fig.S2).


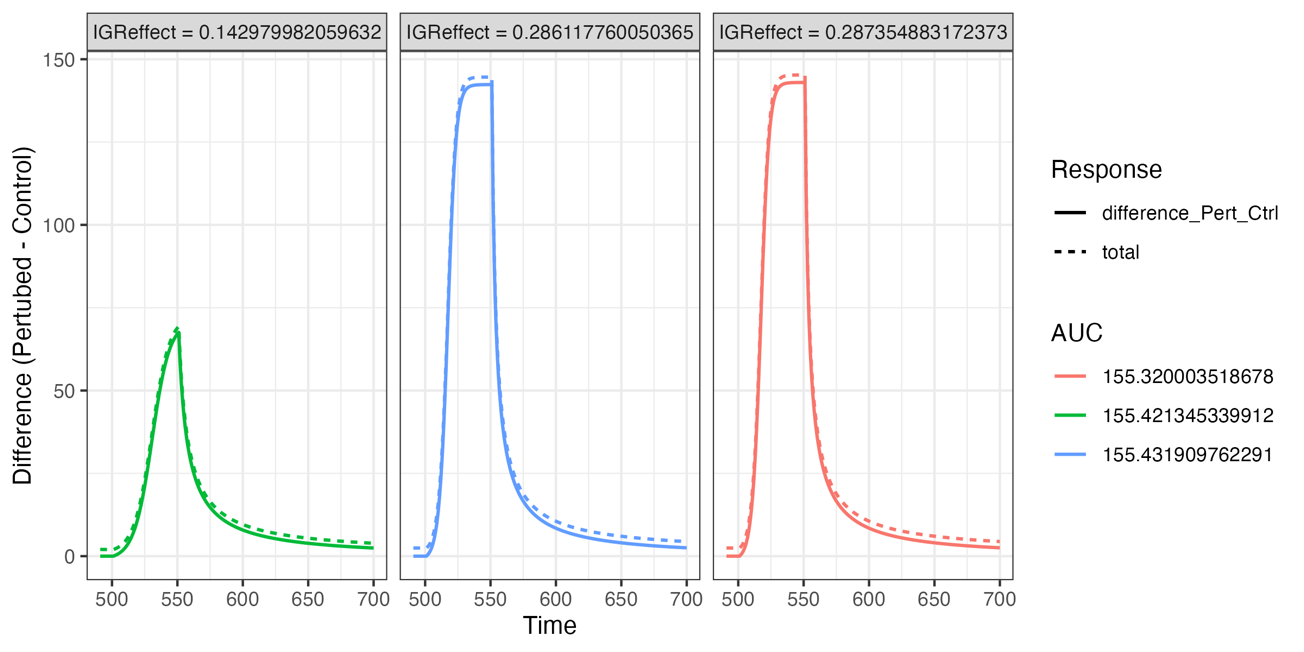


**Box FigS. 1:** Exemplary time series of three species which vary in their fundamental responses. Solid lines show the biomass difference between disturbed and control conditions, while dashed lines represent the summed biomass in control and treatment. The realised response corresponds to the integrated area under the solid curve, scaled by the total sum (AUC values in the legend). Across the three panels, the fundamental responses (IGReffect) differ in magnitude, yet the realised responses converge to similar values because the numerator (difference) is limited by the denominator (sum).


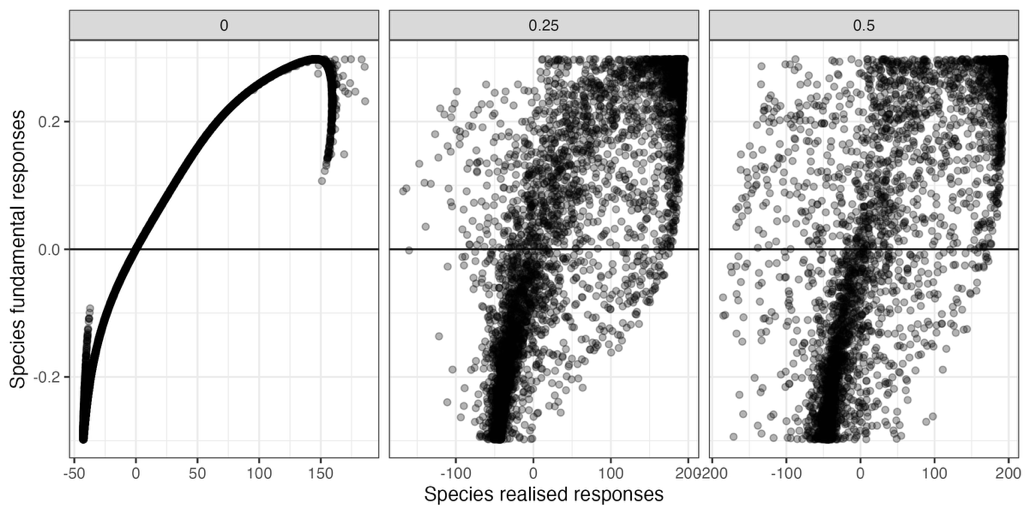


**Box FigS. 2:** Relationship between species realised responses and fundamental species responses over a gradient of interspecific interaction strength (as indicated by different facets). One point represents one species. Realised responses are standardised by the sum of control and disturbed species biomass. This scaling effect explains why the realised response–fundamental response relationship is S-shaped: differences in growth rate translate non-linearly into realised community outcomes once biomass constraints are accounted for.

It is important to clearly distinguish that the fundamental response reflects an absolute difference in rates, describing the change in population growth in a disturbed environment relative to an undisturbed control, whereas the realized response represents an integrated, normalized difference in standing biomass (N), capturing the cumulative deviation in absolute biomass over time. While fundamental responses can vary widely in magnitude - reflecting differences in intrinsic growth rate - realised responses saturate as the relative biomass difference approaches the plateau imposed by standardisation. Thus, realised responses capture the effective outcome of disturbances by integrating both growth performance and biomass constraints.

------------------------------------------------ End of Appendix Box 1 -------------------------------------------------------
