# Supplementary material for: Species Interactions Determine the Importance of Response Diversity for Community Stability to Pulse Disturbances
Source: Ecol Lett. 2025 Dec 31;29(1):e70299. doi: 10.1111/ele.70299 (PMC12755191; doi:10.1111/ele.70299)
Supplement: Supplementary file 2 — Appendix S2: ele70299‐sup‐0002‐AppendixS2.docx. Figure S1: PRISMA Flow for extending meta‐analysis. Source: Page MJ, et al. BMJ 2021; 372:n71. doi: 10.1136/bmj.n71. The original meta‐analysis was performed in 2018 based on a search at the Web of Science (www.webofknowledge.com/WOS, assessed April 3rd, 2018). [file ELE-29-0-s001.docx]

| **Appendix S2**  **Journal: Ecology Letters**  **Article Type: Letter**  **Title:** Species interactions determine the importance of response diversity for community stability to pulse disturbances  **Authors:** Charlotte Kunze, Owen L. Petchey, Shyamolina Ghosh, Helmut Hillebrand   \| **caseID** \| **resp.cat** \| **studyID** \| **ref** \| **spec.inf** \| **system** \| \| --- \| --- \| --- \| --- \| --- \| --- \| \| CK002_1 \| abundance \| CK002 \| (Sarmento et al., 2013) \| taxa \| marine \| \| CK002_2 \| abundance \| CK002 \| (Sarmento et al., 2013) \| taxa \| marine \| \| CK006_1 \| biomass \| CK006 \| (Wardle and Jonsson, 2014) \| species \| terrestrial \| \| CK006_2 \| biomass \| CK006 \| (Wardle and Jonsson, 2014) \| species \| terrestrial \| \| CK006_3 \| biomass \| CK006 \| (Wardle and Jonsson, 2014) \| species \| terrestrial \| \| CK006_4 \| biomass \| CK006 \| (Wardle and Jonsson, 2014) \| species \| terrestrial \| \| CK006_5 \| biomass \| CK006 \| (Wardle and Jonsson, 2014) \| species \| terrestrial \| \| CK006_6 \| biomass \| CK006 \| (Wardle and Jonsson, 2014) \| species \| terrestrial \| \| CK006_7 \| biomass \| CK006 \| (Wardle and Jonsson, 2014) \| species \| terrestrial \| \| CK006_8 \| biomass \| CK006 \| (Wardle and Jonsson, 2014) \| species \| terrestrial \| \| CK006_9 \| biomass \| CK006 \| (Wardle and Jonsson, 2014) \| species \| terrestrial \| \| CK015_7 \| abundance \| CK015 \| (Hua and Relyea, 2014) \| species \| freshwater \| \| CK015_*9* \| abundance \| CK015 \| (Hua and Relyea, 2014) \| species \| freshwater \| \| CK015_12 \| abundance \| CK015 \| (Hua and Relyea, 2014) \| species \| freshwater \| \| CK015_13 \| abundance \| CK015 \| (Hua and Relyea, 2014) \| species \| freshwater \| \| CK015_14 \| abundance \| CK015 \| (Hua and Relyea, 2014) \| species \| freshwater \| \| CK015_15 \| abundance \| CK015 \| (Hua and Relyea, 2014) \| species \| freshwater \| \| CK015_17 \| abundance \| CK015 \| (Hua and Relyea, 2014) \| species \| freshwater \| \| CK015_21 \| abundance \| CK015 \| (Hua and Relyea, 2014) \| species \| freshwater \| \| CK015_25 \| abundance \| CK015 \| (Hua and Relyea, 2014) \| species \| freshwater \| \| CK015_26 \| abundance \| CK015 \| (Hua and Relyea, 2014) \| species \| freshwater \| \| CK015_27 \| abundance \| CK015 \| (Hua and Relyea, 2014) \| species \| freshwater \| \| CK019_1 \| abundance \| CK019 \| (Egres et al., 2012) \| species \| marine \| \| CK019_2 \| abundance \| CK019 \| (Egres et al., 2012) \| species \| marine \| \| CK019_3 \| abundance \| CK019 \| (Egres et al., 2012) \| species \| marine \| \| CK021_1 \| abundance \| CK021 \| (Botter-Carvalho et al., 2011) \| species \| marine \| \| CK027_2 \| biomass \| CK027 \| (Richardson et al., 2010) \| taxa \| terrestrial \| \| CK032_1 \| abundance \| CK032 \| (Forrest and Arnott, 2006) \| species \| freshwater \| \| CK032_2 \| abundance \| CK032 \| (Forrest and Arnott, 2006) \| species \| freshwater \| \| CK041_1 \| abundance \| CK041 \| (Thrush et al., 2003) \| species \| marine \| \| CK041_5 \| abundance \| CK041 \| (Thrush et al., 2003) \| species \| marine \| \| CK041_7 \| abundance \| CK041 \| (Thrush et al., 2003) \| species \| marine \| \| CK041_9 \| abundance \| CK041 \| (Thrush et al., 2003) \| species \| marine \| \| CK043_1 \| abundance \| CK043 \| (Matthaei et al., 1996) \| species \| freshwater \| \| CK043_2 \| abundance \| CK043 \| (Matthaei et al., 1996) \| species \| freshwater \| \| CK045_1 \| abundance \| CK045 \| (Kreutzweiser and Thomas, 1995) \| species \| freshwater \| \| CK045_2 \| abundance \| CK045 \| (Kreutzweiser and Thomas, 1995) \| species \| freshwater \| \| CK045_3 \| abundance \| CK045 \| (Kreutzweiser and Thomas, 1995) \| species \| freshwater \| \| CK045_4 \| abundance \| CK045 \| (Kreutzweiser and Thomas, 1995) \| species \| freshwater \| \| CK045_5 \| abundance \| CK045 \| (Kreutzweiser and Thomas, 1995) \| species \| freshwater \| \| CK045_6 \| abundance \| CK045 \| (Kreutzweiser and Thomas, 1995) \| species \| freshwater \| \| CK045_7 \| abundance \| CK045 \| (Kreutzweiser and Thomas, 1995) \| species \| freshwater \| \| CK045_8 \| abundance \| CK045 \| (Kreutzweiser and Thomas, 1995) \| species \| freshwater \| \| CK049_1 \| abundance \| CK049 \| (Olsen et al., 2007) \| species \| freshwater \| \| CK049_2 \| abundance \| CK049 \| (Olsen et al., 2007) \| species \| freshwater \| \| CK053_1 \| abundance \| CK053 \| (Kröger et al., 2006) \| species \| marine \| \| CK056_1 \| abundance \| CK056 \| (Bolam et al., 2004) \| species \| marine \| \| CK056_2 \| abundance \| CK056 \| (Bolam et al., 2004) \| species \| marine \| \| CK056_3 \| abundance \| CK056 \| (Bolam et al., 2004) \| species \| marine \| \| CK056_4 \| abundance \| CK056 \| (Bolam et al., 2004) \| species \| marine \| \| CK059_1 \| abundance \| CK059 \| (Cristoni et al., 2004)c \| species \| marine \| \| CK059_2 \| abundance \| CK059 \| (Cristoni et al., 2004) \| species \| marine \| \| CK059_3 \| abundance \| CK059 \| (Cristoni et al., 2004) \| species \| marine \| \| CK062_1 \| abundance \| CK062 \| (Dernie et al., 2003) \| species \| marine \| \| CK062_2 \| abundance \| CK062 \| (Dernie et al., 2003) \| species \| marine \| \| CK066_1 \| abundance \| CK066 \| (Spawn et al., 1997) \| species \| freshwater \| \| CK066_2 \| abundance \| CK066 \| (Spawn et al., 1997) \| species \| freshwater \| \| CK066_3 \| abundance \| CK066 \| (Spawn et al., 1997) \| species \| freshwater \| \| CK066_4 \| abundance \| CK066 \| (Spawn et al., 1997) \| species \| freshwater \| \| CK066_5 \| abundance \| CK066 \| (Spawn et al., 1997) \| species \| freshwater \| \| CK067_1 \| biomass \| CK067 \| (Brosnan and Crumrine, 1994) \| species \| marine \| \| CK069_1 \| biomass \| CK069 \| (Kennelly and Underwood, 1993) \| species \| marine \| \| CK069_2 \| biomass \| CK069 \| (Kennelly and Underwood, 1993) \| species \| marine \| \| CK069_3 \| biomass \| CK069 \| (Kennelly and Underwood, 1993) \| species \| marine \| \| CK069_4 \| biomass \| CK069 \| (Kennelly and Underwood, 1993) \| species \| marine \| \| CK069_5 \| biomass \| CK069 \| (Kennelly and Underwood, 1993) \| species \| marine \| \| CK069_6 \| biomass \| CK069 \| (Kennelly and Underwood, 1993) \| species \| marine \| \| CK069_7 \| biomass \| CK069 \| (Kennelly and Underwood, 1993) \| species \| marine \| \| CK069_8 \| biomass \| CK069 \| (Kennelly and Underwood, 1993) \| species \| marine \| \| CK074_1 \| abundance \| CK074 \| (Vander Vorste et al., 2016) \| species \| freshwater \| \| CK074_2 \| abundance \| CK074 \| (Vander Vorste et al., 2016) \| species \| freshwater \| \| CK075_1 \| abundance \| CK075 \| (Zajac and Whitlatch, 2003) \| species \| freshwater \| \| CK080_1 \| biomass \| CK080 \| (McClanahan et al., 2001) \| species \| marine \| \| CK080_1 \| biomass \| CK080 \| (McClanahan et al., 2001) \| taxa \| marine \| \| CK080_2 \| biomass \| CK080 \| (McClanahan et al., 2001) \| species \| marine \| \| CK080_2 \| biomass \| CK080 \| (McClanahan et al., 2001) \| taxa \| marine \| \| CK081_1 \| abundance \| CK081 \| (Syms and Jones, 2000) \| species \| marine \| \| CK081_2 \| abundance \| CK081 \| (Syms and Jones, 2000) \| species \| marine \| \| CK081_3 \| abundance \| CK081 \| (Syms and Jones, 2000) \| species \| marine \| \| CK081_4 \| abundance \| CK081 \| (Syms and Jones, 2000) \| species \| marine \| \| CK081_5 \| abundance \| CK081 \| (Syms and Jones, 2000) \| species \| marine \| \| HH001_34 \| biomass \| HH001 \| (Hillebrand et al., 2018) \| species \| freshwater \| \| HH001_35 \| biomass \| HH001 \| (Hillebrand et al., 2018) \| species \| freshwater \| \| HH001_36 \| biomass \| HH001 \| (Hillebrand et al., 2018) \| species \| freshwater \| \| HH001_37 \| biomass \| HH001 \| (Hillebrand et al., 2018) \| species \| freshwater \| \| HH001_38 \| biomass \| HH001 \| (Hillebrand et al., 2018) \| species \| freshwater \| \| HH001_39 \| biomass \| HH001 \| (Hillebrand et al., 2018) \| species \| freshwater \| \| HH003_1 \| abundance \| HH003 \| (Biggs et al., 1999) \| species \| freshwater \| \| HH003_3 \| abundance \| HH003 \| (Biggs et al., 1999) \| species \| freshwater \| \| HH003_4 \| abundance \| HH003 \| (Biggs et al., 1999) \| species \| freshwater \| \| HH005_1 \| biomass \| HH005 \| (Yáñez et al., 2008) \| species \| marine \| \| HH005_2 \| biomass \| HH005 \| (Yáñez et al., 2008) \| species \| marine \| \| HH006_4 \| abundance \| HH006 \| (Norkko et al., 2002) \| species \| marine \| \| HH006_5 \| abundance \| HH006 \| (Norkko et al., 2002) \| species \| marine \| \| HH006_6 \| abundance \| HH006 \| (Norkko et al., 2002) \| species \| marine \| \| HH009_1 \| abundance \| HH009 \| (Sparks-McConkey and Watling, 2001) \| species \| marine \| \| HH011_1 \| abundance \| HH011 \| (Shiels et al., 2010) \| species \| terrestrial \| \| HH011_2 \| abundance \| HH011 \| (Shiels et al., 2010) \| species \| terrestrial \| \| HH011_3 \| abundance \| HH011 \| (Shiels et al., 2010) \| species \| terrestrial \| \| HH014_1 \| abundance \| HH014 \| (Williams et al., 2001) \| species \| terrestrial \| \| HH018_1 \| abundance \| HH018 \| (Otitoloju et al., 2007) \| species \| marine \| \| CK082_1 \| abundance \| CK082 \| (Mondragón-Camarillo et al., 2020) \| species \| terrestrial \| \| CK083_1 \| abundance \| CK083 \| (Gandiaga and Moreau, 2019) \| taxa \| terrestrial \| \| CK083_2 \| abundance \| CK083 \| (Gandiaga and Moreau, 2019) \| taxa \| terrestrial \| \| CK083_3 \| abundance \| CK083 \| (Gandiaga and Moreau, 2019) \| taxa \| terrestrial \| \| CK084_1 \| biomass \| CK084 \| (Polazzo et al., 2022) \| taxa \| freshwater \| \| CK084_2 \| biomass \| CK084 \| (Polazzo et al., 2022) \| taxa \| freshwater \| \| CK084_3 \| biomass \| CK084 \| (Polazzo et al., 2022) \| taxa \| freshwater \| \| CK084_4 \| biomass \| CK084 \| (Polazzo et al., 2022) \| taxa \| freshwater \| \| CK084_5 \| biomass \| CK084 \| (Polazzo et al., 2022) \| taxa \| freshwater \| \| CK084_6 \| biomass \| CK084 \| (Polazzo et al., 2022) \| taxa \| freshwater \| \| CK085_1 \| biomass \| CK085 \| (Sun and Arnott, 2024) \| taxa \| freshwater \| \| CK086_1 \| abundance \| CK086 \| (Leduc et al., 2024) \| group \| marine \| \| CK086_2 \| abundance \| CK086 \| (Leduc et al., 2024) \| group \| marine \| \| CK087_1 \| biomass \| CK087 \| (García-Astillero et al., 2024) \| species \| marine \| \| CK087_2 \| biomass \| CK087 \| (García-Astillero et al., 2024) \| species \| marine \| \| CK088_1 \| biomass \| CK088 \| (Ersoy et al., 2019) \| taxa \| freshwater \| \| CK089_1 \| biomass \| CK089 \| (Tait and Schiel, 2018) \| species \| marine \| \| CK089_2 \| biomass \| CK089 \| (Tait and Schiel, 2018) \| species \| marine \| \| CK090_1 \| abundance \| CK090 \| (Guilhermic et al., 2023) \| species \| marine \| \| CK091_1 \| biomass \| CK091 \| (Miao et al., 2022) \| species \| terrestrial \| \| CK092_1 \| abundance \| CK092 \| (Zhou et al., 2019) \| species \| marine \| \| CK092_2 \| abundance \| CK092 \| (Zhou et al., 2019) \| species \| marine \| \| CK092_3 \| abundance \| CK092 \| (Zhou et al., 2019) \| species \| marine \| \| CK093_1 \| abundance \| CK093 \| (Suzuki et al., 2021) \| species \| marine \| \| CK093_2 \| abundance \| CK093 \| (Suzuki et al., 2021) \| species \| terrestrial \| \| CK093_3 \| abundance \| CK093 \| (Suzuki et al., 2021) \| species \| terrestrial \| \| CK093_4 \| abundance \| CK093 \| (Suzuki et al., 2021) \| species \| terrestrial \| \| CK093_6 \| abundance \| CK093 \| (Suzuki et al., 2021) \| species \| terrestrial \| \| CK093_7 \| abundance \| CK093 \| (Suzuki et al., 2021) \| species \| terrestrial \| \| CK093_8 \| abundance \| CK093 \| (Suzuki et al., 2021) \| species \| terrestrial \| \| CK093_9 \| abundance \| CK093 \| (Suzuki et al., 2021) \| species \| terrestrial \| \| CK094_1 \| abundance \| CK094 \| (Pocklington et al., 2019) \| species \| marine \| \| CK094_2 \| abundance \| CK094 \| (Pocklington et al., 2019) \| species \| marine \| \| CK094_3 \| abundance \| CK094 \| (Pocklington et al., 2019) \| species \| marine \| \| CK094_4 \| abundance \| CK094 \| (Pocklington et al., 2019) \| species \| marine \| \| CK095_1 \| biomass \| CK095 \| (Cimon and Cusson, 2018) \| species \| marine \| |
| --- | --- | --- | --- | --- | --- | --- | --- | --- | --- | --- | --- | --- | --- | --- | --- | --- | --- | --- | --- | --- | --- | --- | --- | --- | --- | --- | --- | --- | --- | --- | --- | --- | --- | --- | --- | --- | --- | --- | --- | --- | --- | --- | --- | --- | --- | --- | --- | --- | --- | --- | --- | --- | --- | --- | --- | --- | --- | --- | --- | --- | --- | --- | --- | --- | --- | --- | --- | --- | --- | --- | --- | --- | --- | --- | --- | --- | --- | --- | --- | --- | --- | --- | --- | --- | --- | --- | --- | --- | --- | --- | --- | --- | --- | --- | --- | --- | --- | --- | --- | --- | --- | --- | --- | --- | --- | --- | --- | --- | --- | --- | --- | --- | --- | --- | --- | --- | --- | --- | --- | --- | --- | --- | --- | --- | --- | --- | --- | --- | --- | --- | --- | --- | --- | --- | --- | --- | --- | --- | --- | --- | --- | --- | --- | --- | --- | --- | --- | --- | --- | --- | --- | --- | --- | --- | --- | --- | --- | --- | --- | --- | --- | --- | --- | --- | --- | --- | --- | --- | --- | --- | --- | --- | --- | --- | --- | --- | --- | --- | --- | --- | --- | --- | --- | --- | --- | --- | --- | --- | --- | --- | --- | --- | --- | --- | --- | --- | --- | --- | --- | --- | --- | --- | --- | --- | --- | --- | --- | --- | --- | --- | --- | --- | --- | --- | --- | --- | --- | --- | --- | --- | --- | --- | --- | --- | --- | --- | --- | --- | --- | --- | --- | --- | --- | --- | --- | --- | --- | --- | --- | --- | --- | --- | --- | --- | --- | --- | --- | --- | --- | --- | --- | --- | --- | --- | --- | --- | --- | --- | --- | --- | --- | --- | --- | --- | --- | --- | --- | --- | --- | --- | --- | --- | --- | --- | --- | --- | --- | --- | --- | --- | --- | --- | --- | --- | --- | --- | --- | --- | --- | --- | --- | --- | --- | --- | --- | --- | --- | --- | --- | --- | --- | --- | --- | --- | --- | --- | --- | --- | --- | --- | --- | --- | --- | --- | --- | --- | --- | --- | --- | --- | --- | --- | --- | --- | --- | --- | --- | --- | --- | --- | --- | --- | --- | --- | --- | --- | --- | --- | --- | --- | --- | --- | --- | --- | --- | --- | --- | --- | --- | --- | --- | --- | --- | --- | --- | --- | --- | --- | --- | --- | --- | --- | --- | --- | --- | --- | --- | --- | --- | --- | --- | --- | --- | --- | --- | --- | --- | --- | --- | --- | --- | --- | --- | --- | --- | --- | --- | --- | --- | --- | --- | --- | --- | --- | --- | --- | --- | --- | --- | --- | --- | --- | --- | --- | --- | --- | --- | --- | --- | --- | --- | --- | --- | --- | --- | --- | --- | --- | --- | --- | --- | --- | --- | --- | --- | --- | --- | --- | --- | --- | --- | --- | --- | --- | --- | --- | --- | --- | --- | --- | --- | --- | --- | --- | --- | --- | --- | --- | --- | --- | --- | --- | --- | --- | --- | --- | --- | --- | --- | --- | --- | --- | --- | --- | --- | --- | --- | --- | --- | --- | --- | --- | --- | --- | --- | --- | --- | --- | --- | --- | --- | --- | --- | --- | --- | --- | --- | --- | --- | --- | --- | --- | --- | --- | --- | --- | --- | --- | --- | --- | --- | --- | --- | --- | --- | --- | --- | --- | --- | --- | --- | --- | --- | --- | --- | --- | --- | --- | --- | --- | --- | --- | --- | --- | --- | --- | --- | --- | --- | --- | --- | --- | --- | --- | --- | --- | --- | --- | --- | --- | --- | --- | --- | --- | --- | --- | --- | --- | --- | --- | --- | --- | --- | --- | --- | --- | --- | --- | --- | --- | --- | --- | --- | --- | --- | --- | --- | --- | --- | --- | --- | --- | --- | --- | --- | --- | --- | --- | --- | --- | --- | --- | --- | --- | --- | --- | --- | --- | --- | --- | --- | --- | --- | --- | --- | --- | --- | --- | --- | --- | --- | --- | --- | --- | --- | --- | --- | --- | --- | --- | --- | --- | --- | --- | --- | --- | --- | --- | --- | --- | --- | --- | --- | --- | --- | --- | --- | --- | --- | --- | --- | --- | --- | --- | --- | --- | --- | --- | --- | --- | --- | --- | --- | --- | --- | --- | --- | --- | --- | --- | --- | --- | --- | --- | --- | --- | --- | --- | --- | --- | --- | --- | --- | --- | --- | --- | --- | --- | --- | --- | --- | --- | --- | --- | --- | --- | --- | --- | --- | --- | --- | --- | --- | --- | --- | --- | --- | --- | --- | --- | --- | --- | --- | --- | --- | --- | --- | --- | --- | --- | --- | --- | --- | --- | --- | --- | --- | --- | --- | --- | --- | --- | --- | --- | --- | --- | --- | --- | --- | --- | --- | --- | --- | --- | --- | --- | --- | --- | --- | --- | --- | --- | --- | --- | --- | --- | --- | --- | --- | --- | --- | --- | --- | --- | --- | --- | --- | --- | --- | --- | --- | --- | --- | --- | --- | --- | --- | --- | --- | --- | --- | --- | --- | --- | --- | --- | --- | --- | --- | --- | --- | --- | --- | --- | --- | --- | --- | --- | --- | --- | --- | --- | --- | --- | --- | --- | --- | --- | --- | --- | --- | --- | --- | --- | --- | --- | --- | --- | --- | --- | --- | --- | --- | --- | --- | --- | --- | --- | --- | --- | --- | --- | --- | --- | --- | --- | --- | --- | --- | --- | --- | --- | --- | --- | --- | --- | --- | --- |

**Bibliography**

Biggs, B.J.F., Tuchman, N.C., Lowe, R.L., Stevenson, R.J., 1999. Resource Stress Alters Hydrological Disturbance Effects in a Stream periphyton Community. Oikos 85, 95. https://doi.org/10.2307/3546795

Bolam, S.G., Whomersley, P., Schratzberger, M., 2004. Macrofaunal recolonization on intertidal mudflats: effect of sediment organic and sand content. Journal of Experimental Marine Biology and Ecology 306, 157–180. https://doi.org/10.1016/j.jembe.2004.01.007

Botter-Carvalho, M.L., Carvalho, P.V.V.C., Santos, P.J.P., 2011. Recovery of macrobenthos in defaunated tropical estuarine sediments. Marine Pollution Bulletin 62, 1867–1876. https://doi.org/10.1016/j.marpolbul.2011.04.044

Brosnan, D.M., Crumrine, L.L., 1994. Effects of human trampling on marine rocky shore communities. Journal of Experimental Marine Biology and Ecology 177, 79–97. https://doi.org/10.1016/0022-0981(94)90145-7

Cimon, S., Cusson, M., 2018. Impact of multiple disturbances and stress on the temporal trajectories and resilience of benthic intertidal communities. Ecosphere 9, e02467. https://doi.org/10.1002/ecs2.2467

Cristoni, C., Colangelo, M.A., Ceccherelli, V.U., 2004. Spatial scale and meiobenthic copepod recolonisation: testing the effect of disturbance size in a seagrass habitat. Journal of Experimental Marine Biology and Ecology 298, 49–70. https://doi.org/10.1016/j.jembe.2003.08.005

Dernie, K.M., Kaiser, M.J., Richardson, E.A., Warwick, R.M., 2003. Recovery of soft sediment communities and habitats following physical disturbance. Journal of Experimental Marine Biology and Ecology 285–286, 415–434. https://doi.org/10.1016/S0022-0981(02)00541-5

Egres, A.G., Martins, C.C., Oliveira, V.M.D., Lana, P.D.C., 2012. Effects of an experimental in situ diesel oil spill on the benthic community of unvegetated tidal flats in a subtropical estuary (Paranaguá Bay, Brazil). Marine Pollution Bulletin 64, 2681–2691. https://doi.org/10.1016/j.marpolbul.2012.10.007

Ersoy, Z., Brucet, S., Bartrons, M., Mehner, T., 2019. Short-term fish predation destroys resilience of zooplankton communities and prevents recovery of phytoplankton control by zooplankton grazing. PLoS ONE 14, e0212351. https://doi.org/10.1371/journal.pone.0212351

Forrest, J., Arnott, S.E., 2006. Immigration and zooplankton community responses to nutrient enrichment: a mesocosm experiment. Oecologia 150, 119–131. https://doi.org/10.1007/s00442-006-0490-4

Gandiaga, F., Moreau, G., 2019. How long are thinning-induced resource pulses maintained in plantation forests? Forest Ecology and Management 440, 113–121. https://doi.org/10.1016/j.foreco.2019.02.032

García-Astillero, A., Polazzo, F., Rico, A., 2024. Combined effects of heat waves and pesticide pollution on zooplankton communities: Does the timing of stressor matter? Ecotoxicology and Environmental Safety 282, 116751. https://doi.org/10.1016/j.ecoenv.2024.116751

Guilhermic, C., Nardelli, M.P., Mouret, A., Le Moigne, D., Howa, H., 2023. Short-term response of benthic foraminifera to fine-sediment depositional events simulated in microcosm. Biogeosciences 20, 3329–3351. https://doi.org/10.5194/bg-20-3329-2023

Hillebrand, H., Langenheder, S., Lebret, K., Lindström, E., Östman, Ö., Striebel, M., 2018. Decomposing multiple dimensions of stability in global change experiments. Ecol Lett 21, 21–30. https://doi.org/10.1111/ele.12867

Hua, J., Relyea, R., 2014. Chemical cocktails in aquatic systems: Pesticide effects on the response and recovery of >20 animal taxa. Environmental Pollution 189, 18–26. https://doi.org/10.1016/j.envpol.2014.02.007

Kennelly, S.J., Underwood, A.J., 1993. Geographic consistencies of effects of experimental physical disturbance on understorey species in sublittoral kelp forests in central New South Wales. Journal of Experimental Marine Biology and Ecology 168, 35–58. https://doi.org/10.1016/0022-0981(93)90115-5

Kreutzweiser, D.P., Thomas, D.R., 1995. Effects of a new molt-inducing insecticide, tebufenozide, on zooplankton communities in lake enclosures. Ecotoxicology 4, 307–328. https://doi.org/10.1007/BF00118597

Kröger, K., Gardner, J., Rowden, A., Wear, R., 2006. Recovery of a subtidal soft-sediment macroinvertebrate assemblage following experimentally induced effects of a harmful algal bloom. Mar. Ecol. Prog. Ser. 326, 85–98. https://doi.org/10.3354/meps326085

Leduc, D., Murray, C., Rowden, A.A., Nodder, S.D., Hale, R., Clark, M.R., 2024. Experimental seabed disturbance effects on Chatham Rise deep-sea meiofaunal communities, Southwest Pacific. New Zealand Journal of Marine and Freshwater Research 59, 1388–1421. https://doi.org/10.1080/00288330.2024.2347623

Matthaei, C., Uehlinger, U., Meyer, E., Frutiger, A., 1996. Recolonization by benthic invertebrates after experimental disturbance in a Swiss prealpine river. Freshwater Biology 35, 233–248. https://doi.org/10.1046/j.1365-2427.1996.00496.x

McClanahan, T., McField, M., Huitric, M., Bergman, K., Sala, E., Nyström, M., Nordemar, I., Elfwing, T., Muthiga, N., 2001. Responses of algae, corals and fish to the reduction of macroalgae in fished and unfished patch reefs of Glovers Reef Atoll, Belize. Coral Reefs 19, 367–379. https://doi.org/10.1007/s003380000131

Miao, Y., Zhou, Z., Jiang, M., Song, H., Yan, X., Liu, P., Ji, M., Han, S., Chen, A., Wang, D., 2022. Resistance and Resilience of Nine Plant Species to Drought in Inner Mongolia Temperate Grasslands of Northern China. Applied Sciences 12, 4967. https://doi.org/10.3390/app12104967

Mondragón-Camarillo, L., Zaragoza, S.R., Mendoza-López, Ma.R., Cabirol, N., Macek, M., 2020. Recovery of Soil Protozoan Community Structure Promoted by M. sativa After a Strong Pulse of Hydrocarbon Contamination. Water Air Soil Pollut 231, 283. https://doi.org/10.1007/s11270-020-04618-7

Norkko, A., Thrush, S., Hewitt, J., Cummings, V., Norkko, J., Ellis, J., Funnell, G., Schultz, D., MacDonald, I., 2002. Smothering of estuarine sandflats by terrigenous clay: the role of wind-wave disturbance and bioturbation in site-dependent macrofaunal recovery. Mar. Ecol. Prog. Ser. 234, 23–42. https://doi.org/10.3354/meps234023

Olsen, D.A., Matthaei, C.D., Townsend, C.R., 2007. Patch history, invertebrate patch dynamics and heterogeneous community composition: perspectives from a manipulative stream experiment. Mar. Freshwater Res. 58, 307. https://doi.org/10.1071/MF06202

Otitoloju, A.A., Are, T., Junaid, K.A., 2007. Recovery assessment of a refined-oil impacted and fire ravaged mangrove ecosystem. Environ Monit Assess 127, 353–362. https://doi.org/10.1007/s10661-006-9285-7

Pocklington, J.B., Keough, M.J., O’Hara, T.D., Bellgrove, A., 2019. The Influence of Canopy Cover on the Ecological Function of A Key Autogenic Ecosystem Engineer. Diversity 11, 79. https://doi.org/10.3390/d11050079

Polazzo, F., Roth, S.K., Hermann, M., Mangold‐Döring, A., Rico, A., Sobek, A., Van den Brink, P.J., Jackson, M.C., 2022. Combined effects of heatwaves and micropollutants on freshwater ecosystems: Towards an integrated assessment of extreme events in multiple stressors research. Global Change Biology 28, 1248–1267. https://doi.org/10.1111/gcb.15971

Richardson, B.A., Richardson, M.J., González, G., Shiels, A.B., Srivastava, D.S., 2010. A Canopy Trimming Experiment in Puerto Rico: The Response of Litter Invertebrate Communities to Canopy Loss and Debris Deposition in a Tropical Forest Subject to Hurricanes. Ecosystems 13, 286–301. https://doi.org/10.1007/s10021-010-9317-6

Sarmento, V.C., Barreto, A.F.S., Santos, P.J.P., 2013. Recovery of meiofauna following a short-term disturbance on coral reefs. Biodivers Conserv 22, 2645–2663. https://doi.org/10.1007/s10531-013-0548-7

Shiels, A.B., Zimmerman, J.K., García‐Montiel, D.C., Jonckheere, I., Holm, J., Horton, D., Brokaw, N., 2010. Plant responses to simulated hurricane impacts in a subtropical wet forest, Puerto Rico. Journal of Ecology 98, 659–673. https://doi.org/10.1111/j.1365-2745.2010.01646.x

Sparks-McConkey, P.J., Watling, L., 2001. Effects on the ecological integrity of a soft-bottom habitat from a trawling disturbance. Hydrobiologia 456, 73–85.

Spawn, R.L., Hoagland, K.D., Siegfried, B.D., 1997. Effects of Alachlor on an Algal Community from a Midwestern Agricultural Stream. Environmental Toxicology and Chemistry 16, 785–793. https://doi.org/DOI:%252010.1897/1551-5028(1997)016%253C0785:

Sun, X., Arnott, S.E., 2024. Timing determines zooplankton community responses to multiple stressors. Global Change Biology 30, e17358. https://doi.org/10.1111/gcb.17358

Suzuki, M., Karukome, T., Fujihira, K., Mitsugi, M., Hisamoto, Y., 2021. Clear‐cutting triggers regeneration of abandoned secondary forests but risks alternative successional trajectories with high deer density. Applied Vegetation Science 24, e12596. https://doi.org/10.1111/avsc.12596

Syms, C., Jones, G.P., 2000. Disturbance, Habitat Structure, and the Dynamics of a Coral-Reef Fish Community. Ecology 81, 2741–2729.

Tait, L.W., Schiel, D.R., 2018. Ecophysiology of Layered Macroalgal Assemblages: Importance of Subcanopy Species Biodiversity in Buffering Primary Production. Front. Mar. Sci. 5, 444. https://doi.org/10.3389/fmars.2018.00444

Thrush, S.F., Hewitt, J.E., Norkko, A., Cummings, V.J., Funnell, G.A., 2003. MACROBENTHIC RECOVERY PROCESSES FOLLOWING CATASTROPHIC SEDIMENTATION ON ESTUARINE SANDFLATS. Ecological Applications 13, 1433–1455. https://doi.org/10.1890/02-5198

Vander Vorste, R., Malard, F., Datry, T., 2016. Is drift the primary process promoting the resilience of river invertebrate communities? A manipulative field experiment in an intermittent alluvial river. Freshwater Biology 61, 1276–1292. https://doi.org/10.1111/fwb.12658

Wardle, D.A., Jonsson, M., 2014. Long-term resilience of above- and belowground ecosystem components among contrasting ecosystems. Ecology 95, 1836–1849.

Williams, M.R., Abbott, I., Liddelow, G.L., Vellios, C., Wheeler, I.B., Mellican, A.E., 2001. Recovery of bird populations after clearfelling of tall open eucalypt forest in Western Australia. Journal of Applied Ecology 38, 910–920. https://doi.org/10.1046/j.1365-2664.2001.00645.x

Yáñez, B., Carballo, J.L., Olabarria, C., Barrón, J.J., 2008. Recovery of macrobenthic assemblages following experimental sand burial. OCEANOLOGIA 50, 391–420.

Zajac, R., Whitlatch, R.B., 2003. Community and population-level responses to disturbance in a sandflat community. Journal of Experimental Marine Biology and Ecology 294, 101–125. https://doi.org/10.1016/S0022-0981(03)00262-4

Zhou, Z., Li, X., Chen, L., Li, B., Wang, C., Guo, J., Shi, P., Yang, L., Liu, B., Song, B., 2019. Effects of diesel oil spill on macrobenthic assemblages at the intertidal zone: A mesocosm experiment in situ. Marine Environmental Research 152, 104823. https://doi.org/10.1016/j.marenvres.2019.104823

**Previous studies**

**Identification of new studies via databases and registers**

Studies included in previous version of review (n = 98)

Records identified from WOS:

Databases (n = 467)

Records removed *before screening*:

Duplicate records removed (n = 6)

Records removed for other reasons (n = 1)

**Identification**

Records screened

(n = 97)

Records excluded**

(n = 363)

Reports not retrieved

(n = 7)

Reports sought for retrieval

(n = 21)

**Screening**

Reports assessed for eligibility

(n = 14)

New experiments included in review

(n = 30)

**Included**

Total studies included in review

(n = 134)

*

**Figure S1:** PRISMA Flow for extending meta-analysis. Source: Page MJ, et al. BMJ 2021; 372:n71. doi: 10.1136/bmj.n71. The original meta-analysis was performed in 2018 based on a search at the Web of Science (www.webofknowledge.com/WOS, assessed April 3rd, 2018).
